# Parallel-in-Time Preconditioning for Time-Dependent Variational Mean Field Games

**DOI:** 10.1007/s10915-026-03501-7

**Published:** 2026-10-12

**Authors:** Heidi Wolles Ljósheim, Dante Kalise, John W. Pearson, Francisco J. Silva

**Affiliations:** 1 School of Mathematics and Maxwell Institute for Mathematical Sciences, The University of Edinburgh, James Clerk Maxwell Building, The King’s Buildings, Edinburgh, EH9 3FD UK; 2 Department of Mathematics, Imperial College London, South Kensington Campus, London, SW7 2AZ UK; 3 Institut de recherche XLIM-DMI, UMR 7252 CNRS, Faculté des Sciences et Techniques, Université de Limoges, 87060 Limoges, France

**Keywords:** Mean field games, Preconditioned iterative method, Parallel-in-time solver, Primal–dual algorithm, PDE-constrained optimization

## Abstract

We study the numerical approximation of a time-dependent variational mean field game system with local couplings and either periodic or Neumann boundary conditions. Following a variational approach, we employ a finite-difference discretization and solve the resulting finite-dimensional optimization problem using the Chambolle–Pock primal–dual algorithm. As this involves computing proximal operators and solving ill-conditioned linear systems at each iteration, we embed within our solver a general class of parallel-in-time preconditioners based on suitably-chosen diagonalization techniques, applied using fast discrete transforms. These enable efficient, scalable iterative solvers for each linear system, with robustness across a wide range of viscosities. We further develop fast solvers for the resulting ill-conditioned systems arising at each time step, using exact recursive schemes for structured grids while allowing for other geometries. Numerical experiments confirm the improved performance and parallel scalability of our approach.

## Introduction

Mean field game (MFG) theory has been introduced independently by Lasry and Lions [1–3] and by Huang et al. [4]. The aim of MFGs is to model the asymptotic behaviour of equilibria of symmetric differential games as the number of players tends to infinity. We refer the reader to [5–8] for background in MFG theory, the numerical approximation of equilibria, probabilistic techniques, and applications.

In this work we consider the following time-dependent MFG system with local couplings and periodic boundary conditions:

**Figure:**
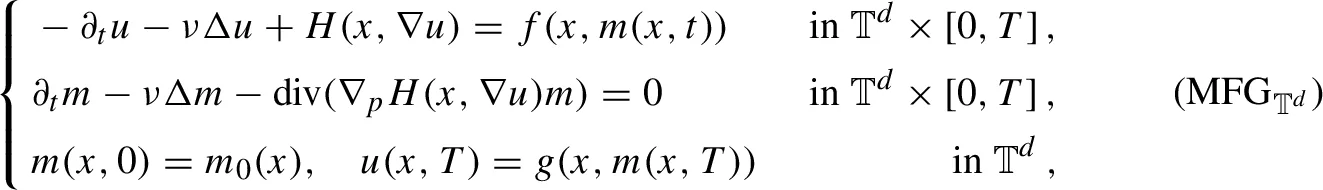

where $\mathbb {T}^{d}$ is the *d*-dimensional torus, T>0, the viscosity $\nu \ge 0$, $H:\mathbb {T}^{d}\times \mathbb {R}^d\rightarrow \mathbb {R}$ is jointly continuous, convex, and differentiable with respect to its second variable, *f*, $g: \mathbb {T}^{d}\times [0,+\infty )\rightarrow \mathbb {R}$ are continuous functions, and $m_0 \in L^1(\mathbb {T}^{d})$ satisfies $m_0 \ge 0$ a.e. and $\int _{\mathbb {T}^{d}}m_0(x) \,dx=1$, hence representing a probability density function. Given an open bounded domain $\emptyset \ne \Omega \subseteq \mathbb {R}^{d}$, we also consider a variant of $(\textrm{MFG}_{\mathbb {T}^{d}})$ with the following additional Neumann boundary conditions:

$$\begin{aligned} \nabla u(x)\cdot n(x)&= 0 \;\text { on }\; \partial \Omega \times [0,T], \\ \left( \nu \nabla m+\nabla _p H(x,\nabla u)m\right) \cdot n(x)&=0 \;\text { on } \;\partial \Omega \times [0,T], \end{aligned}$$

where now $H:\overline{\Omega }\times \mathbb {R}^d\rightarrow \mathbb {R}$, and *n*(*x*) denotes a unit outward normal vector at $x\in \partial \Omega$. We also define *f*, $g: \overline{\Omega }\times [0,+\infty )\rightarrow \mathbb {R}$, $m_0:\Omega \rightarrow \mathbb {R}$, with support contained in $\overline{\Omega }$, and we replace ${\mathbb {T}^d}$ by Ω wherever it appears in the original system ($\textrm{MFG}_{\mathbb {T}^{d}}$), denoting this new system by $({\textrm{MFG}}_{\Omega })$.

Under various assumptions on the Hamiltonian *H*, the coupling functions *f* and *g*, and the initial distribution $m_0$, the existence and uniqueness of solutions to ($\textrm{MFG}_{\mathbb {T}^{d}}$) have been studied in several works, starting from [2, 3], using fixed point techniques. Instead of providing an exhaustive list of references, we refer the reader to [9, 10] and the references therein for a detailed account on the well-posedness of ($\textrm{MFG}_{\mathbb {T}^{d}}$). Regarding system ($\text {MFG}_{\Omega })$, we refer the reader to [11, 12] for existence and uniqueness results and to [13] for the study of the associated master equation.

The numerical approximation of MFG systems has been an active research topic during the last decade. We refer the reader to [14–16] and the references therein for an overview of this subject. With classical sources employing the Newton method [14, 17], recent numerical solution methods for MFGs include an augmented Lagrangian method [18, 19], a primal–dual method [20, 21], and fictitious play, or, the generalized conditional gradient method [22–24]. As noticed in [3], systems of the form ($\textrm{MFG}_{\mathbb {T}^{d}}$)–($\text {MFG}_{\Omega })$ admit a variational formulation, i.e., they correspond to the optimality system of an underlying optimization problem. Taking advantage of this structure, a promising approach in numerical methods for MFGs is to discretize the underlying partial differential equation (PDE) and treat it as the optimality system of a finite-dimensional problem, allowing the use of convex optimization techniques to calculate equilibria (see, e.g., [18, 20, 21, 23, 25–29]).

As in [20, 21], we focus on the application of the primal–dual method introduced in [30] applied to a finite-difference discretization of ($\textrm{MFG}_{\mathbb {T}^{d}}$). We also consider a similar discretization of the underlying variational problem associated with ($\text {MFG}_{\Omega })$. At each iteration, the primal–dual method requires the computation of two proximal operators [31, 32]. In this work we restrict our attention to Hamiltonians of the form

1 $$\begin{aligned} \mathbb {T}^{d}\times \mathbb {R}^{d}\ni (x,p)\mapsto H(x,p)=\frac{1}{q'}|p|^{q'}\in \mathbb {R}, \end{aligned}$$

with $q'\in (1,+\infty )$ and |*p*| the Euclidean norm of *p*, for two reasons. Firstly, Hamiltonians of this form are a natural choice in control-related applications, as they penalize large velocities and are therefore well suited to crowd motion models; we refer the reader to the numerical tests in Sect. 5 for concrete examples. Secondly, the associated proximal operators admit a straightforward computation, although the methodology can be extended to more general Hamiltonians along the lines of [33]. Having fixed this class of Hamiltonians, we concentrate our efforts on providing an efficient solution method for the linear systems arising in the primal–dual iterations.

The purpose of this paper is to improve the performance of the solution method in [21] by considering a novel set of preconditioners for the linear systems arising in the primal–dual algorithm. Let $\mathcal {D}$ denote the discrete version of the differential operator $(\partial _t - \nu \Delta , \text {div})$, then the dual proximal operator inevitably involves applying the inverse of a matrix of the form $\mathcal {D}\mathcal {D}^*$. Since non-zero viscosity MFGs admit a second-order differential operator, this leads to a fourth-order operator in the matrix $\mathcal {D}\mathcal {D}^*$. The condition number for a similar (simpler) fourth-order operator was observed to scale as $\mathcal {O}(h^{-4})$ when the discretization parameter *h* tends to zero in [34], showing the necessity of preconditioning in order to obtain accurate results in a reasonable time.

A number of techniques have been tested to improve the convergence of Krylov subspace solvers for systems of the form $\mathcal {D}\mathcal {D}^*$ arising from MFGs, most popularly multigrid methods in [19, 21, 34]. These show reasonable results which generally improve when increasing the diffusion ν. More elaborate preconditioning strategies include applying a temporal transformation that produces a block-diagonalizable preconditioner [19]. However, we note that [19] deals with operators from a temporal discretization similar to continuous Galerkin time-stepping, and the alternating direction method of multipliers (ADMM) is employed as the solution method. This leads to a matrix involving transposes of these operators, although similar preconditioning principles apply.

In this work, we propose a class of fully parallel-in-time preconditioners based on the theory of (block-) diagonalizing in time using (real-valued) discrete Fourier transforms (DFTs), for systems arising when solving variational mean field games using the Chambolle–Pock algorithm. This allows a parallel implementation, which is currently the main advantage of the primal–dual algorithm. Moreover, the preconditioner is exact for zero viscosity, as observed in [35]. In comparison, a second-order method like the Newton method will generally not permit a parallel-in-time preconditioner unless the equations are approximated in a way that yields time-uniform blocks. Indeed, a Newton linearization will lead to very different linear systems at every iteration, which is not the case with the Chambolle–Pock algorithm, meaning that we may re-use a preconditioner from one Chambolle–Pock iteration to another. Moreover, the Newton method can be shown to struggle with small viscosity regimes as *m* may be negative at some iterations [34]. This behaviour may be remedied by a method such as warm-starting the algorithm with solutions of problems involving increasingly small viscosities, or by using an intermediate solution found with a first-order method.

The preconditioning strategy is complete with a number of different options for solving the system involving the fourth-order differential operator which arises at each time step: we construct exact recursive linear algebra solvers for the finite-difference schemes with either periodic or Neumann boundary conditions, and have the flexibility to defer to LU factorization and algebraic multigrid (AMG) methods for more general meshes. In a number of numerical experiments, the preconditioners show robustness with respect to the viscosity parameter ν, and good scaling with respect to their parallelization capabilities.

The key contributions of this paper are summarised as follows:

- A framework for the preconditioned iterative solution of systems arising from variational MFGs, that allows the application of different parallelization strategies across the time variable. Between these strategies, rapid convergence is achieved in settings with both large and small viscosity parameters.
- The incorporation of effective strategies for preconditioning the matrices across the spatial variables arising at every time step, including a recursive, block DFT linear solver for certain settings.
- The practical and robust numerical solution of very large MFG systems, previously untested in the literature to our knowledge, in particular systems within the numerically-challenging regime of small but non-zero viscosities.
- A fully parallel implementation for different MFGs, leading to convincing strong and weak scaling results for our preconditioned iterative solver.
- The preconditioners are applicable beyond the Chambolle–Pock algorithm as systems involving the matrix $\mathcal {D}\mathcal {D}^*$ may arise in a number of solution algorithms, including when solving normal equations.

This paper is structured as follows: In Sect. 2, we introduce some standard notation and recall the finite-difference scheme for ($\textrm{MFG}_{\mathbb {T}^{d}}$) as originally proposed in [17]. We extend this to the variational formulation that we examine, including the setting with Neumann boundary conditions. Section 3 is devoted to the primal–dual algorithm introduced in [30], and we discuss its application to the variational problem. In Sect. 4, we present our new preconditioning strategy. In Sect. 5, we present numerical results for two well-known test problems, accompanied by a number of parallel scaling tests.

## Finite-Difference Discretizations

In this section, we first recall the finite-difference discretization of ($\textrm{MFG}_{\mathbb {T}^{d}}$) introduced in [17] and provide a slight variant to deal with the Neumann boundary conditions in ($\text {MFG}_{\Omega })$. For the sake of simplicity, we will focus on the two-dimensional case d=2 and, as mentioned in the introduction, we consider Hamiltonians of the form (1).

Consider a rectangular domain $\Omega =[a,b]\times [c,d]$, the height and width of which for periodic boundary conditions correspond to the circumferences of the torus. Given positive integers $N_t$, $N_x$, $N_y$ and setting $\Delta t=T/N_t$, $h=(\Delta x, \Delta y)=((b-a)/N_x,(d-c)/N_y)$, we define the time and space grids, respectively,

$$\begin{aligned} T_{\Delta t}&:= \{t_k = k\Delta t\; ; \; k=0,\dots ,N_t\}, \\ \mathbb {T}_{h}^{2}&:= \{x_{i,j}=(a+i\Delta x,c+j\Delta y) \; ; \; i=0,1,\ldots ,N_x-1,\;j=0,1,\ldots ,N_y-1\}. \end{aligned}$$

For a discrete scalar field $\alpha :\mathbb {T}_{h}^{2}\times T_{\Delta t} \rightarrow \mathbb {R}$, indexed as $\alpha _{i,j}^k$, we define for $T_{\Delta t}$ and $\mathbb {T}_{h}^{2}$ the finite-difference operators

$$\begin{aligned}&(D_1 \alpha )^k_{i,j} := \frac{\alpha ^k_{i+1,j}-\alpha ^k_{i,j}}{\Delta x},\quad (D_2 \alpha )^k_{i,j} := \frac{\alpha ^k_{i,j+1}-\alpha ^k_{i,j}}{\Delta y},\quad (D_t \alpha )^k_{i,j} := \frac{\alpha ^{k+1}_{i,j}-\alpha ^k_{i,j}}{\Delta t}\,,\\&\text {and } (\Delta _h \alpha )^k_{i,j} := \frac{\alpha _{i+1,j}^{k}-2\alpha ^k_{i,j}+\alpha _{i-1,j}^{k}}{(\Delta x)^2}+ \frac{\alpha _{i,j+1}^{k}-2\alpha ^k_{i,j}+\alpha _{i,j-1}^{k}}{(\Delta y)^2}, \end{aligned}$$

where for periodic boundary conditions the algebraic operations over the indices *i* and *j* are taken modulo $N_x$ and $N_y$, respectively. These operators correspond to discrete first-order derivatives in space and time, and the discrete Laplacian operator, respectively. We also set

$$ [D_h \alpha ]_{i,j}^{k}:= ((D_1 \alpha )^k_{i,j},(D_1 \alpha )^k_{i-1,j}, (D_2 \alpha )^k_{i,j},(D_2 \alpha )^k_{i,j-1})\in \mathbb {R}^4 $$

and, given $w:\mathbb {T}_{h}^{2}\rightarrow \mathbb {R}^4$ indexed as $(w^{(1)},w^{(2)},w^{(3)},w^{(4)})_{i,j}$, the discrete divergence operator is given by

$$\begin{aligned} (\textrm{div}_h w)_{i,j}:=(D_1w^{(1)})_{i-1,j}+(D_1w^{(2)})_{i,j}+(D_2w^{(3)})_{i,j-1}+(D_2w^{(4)})_{i,j}. \end{aligned}$$

Let $\mathcal {K}=[0,+\infty )\times (-\infty ,0]\times [0,+\infty )\times (-\infty ,0]$ and, for every $z\in \mathbb {R}^{4}$, set $P_{\mathcal {K}}(z)=\left( [z_1]_{+},[z_{2}]_{-},[z_{3}]_{+},[z_{4}]_{-}\right)$ for its orthogonal projection onto $\mathcal {K}$, where, for $r\in \mathbb {R}$, $[r]_{+}=\max \{r,0\}$ and $[r]_{-}=\min \{0,r\}$.

In [17], the authors propose a finite-difference scheme for $(\textrm{MFG}_{\mathbb {T}^{2}})$ which, in the case of the Hamiltonian (1), takes the form:

**Figure:**
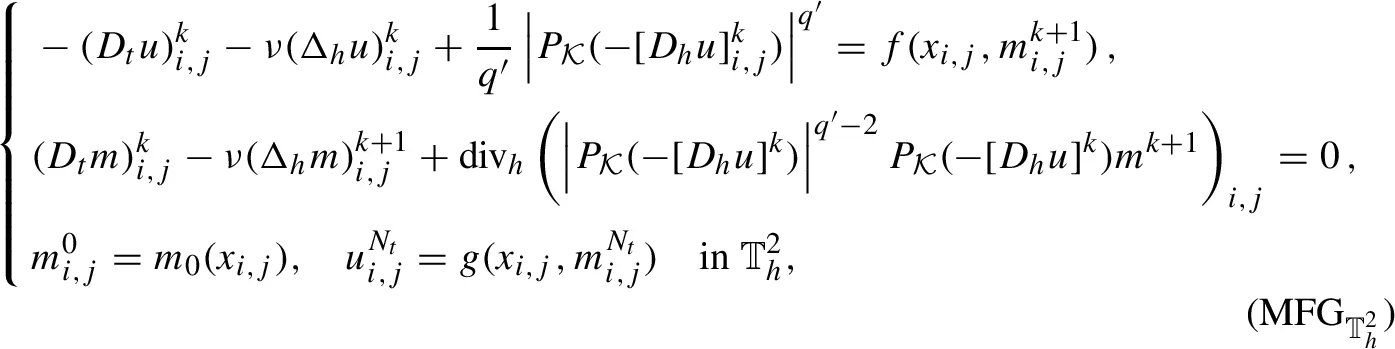

where the first two equations hold in $\mathbb {T}^{2}_{h}\times T_{\Delta t}$. The use of the projection $P_{\mathcal {K}}(-[D_h u]_{i,j}^{k})$ in the numerical Hamiltonian reflects the upwind-type structure of the scheme, in the spirit of [36]. The convergence of solutions to ($\textrm{MFG}_{\mathbb {T}^{2}_{h}}$) towards solutions to $(\textrm{MFG}_{\mathbb {T}^{2}})$ has been established in [37] for classical solutions and in [38] for weak solutions.

For Neumann boundary conditions, we set the discretization parameters $h=(\Delta x,\Delta y)=((b-a)/(N_x+1),(d-c)/(N_y+1))$ such that the internal nodes and the boundary nodes are given by, respectively,

$$\begin{aligned} \Omega _h&:= \{x_{i,j}=(a+i\Delta x,c+j\Delta y) \; ; \; i=1,2,\ldots ,N_{x},\;j=1,2,\ldots ,N_{y}\}, \\ \partial \Omega _{h}&:= \{x_{0,j},x_{N_x+1,j},x_{i,0},x_{i,N_y+1} \; ; \; i=0,1,\ldots ,N_{x}+1,\;j=0,1,\ldots ,N_{y}+1\}. \end{aligned}$$

This definition ensures that the number of unknowns in the space–time grid remains the same, irrespective of boundary conditions. The finite-difference operators in space are now defined only for the internal nodes, not modulo $N_x$ nor $N_y$. The discretization of ($\text {MFG}_{\Omega })$ is equivalent to ($\textrm{MFG}_{\mathbb {T}^{2}_{h}}$) with $\mathbb {T}^2_h$ replaced by $\Omega _h$ and the following additional constraints defined on $\partial \Omega _{h}$:

$$\begin{aligned}&-\nu (D_1 m)^{k+1}_{0,j}+\Phi (u)^k_{0,j,1}m^{k+1}_{0,j} +\Phi (u)^k_{1,j,2}m^{k+1}_{1,j}=0, \\&\nu (D_1 m)^{k+1}_{N_x,j}-\Phi (u)^k_{N_x,j,1}m^{k+1}_{N_x,j} -\Phi (u)^k_{N_x+1,j,2}m^{k+1}_{N_x+1,j}=0, \\&-\nu (D_2 m)^{k+1}_{i,0}+\Phi (u)^k_{i,0,3}m^{k+1}_{i,0} +\Phi (u)^k_{i,1,4}m^{k+1}_{i,1}=0, \\&\nu (D_2 m)^{k+1}_{i,N_y}-\Phi (u)^k_{i,N_y,3}m^{k+1}_{i,N_y}-\Phi (u)^k_{i,N_y+1,4}m^{k+1}_{i,N_y+1}=0, \\&(D_1u)_{0,j}^{k}=(D_1u)_{N_x,j}^{k}=(D_2u)_{i,0}^{k}=(D_2u)_{i,N_y}^{k}=0, \end{aligned}$$

where $\Phi (u)^k_{i,j,\cdot }$ denotes the entries of $\left( \left| P_{\mathcal {K}}(-[D_h u]^{k})\right| ^{q'-2}P_{\mathcal {K}}(-[D_h u]^{k})\right) _{i,j}$ (out of 4). We denote this discretized system by ($\text {MFG}_{\Omega _h})$. The Neumann boundary conditions with mixed indices in the terms Φ(u)m are inspired by the scheme successfully implemented in [39], in the context of more complex MFG problems involving interactions among agents through their control strategies. However, they use fictitious boundary nodes and set all (boundary) terms involving Φ(u) to zero using the boundary information about *u*. This nevertheless leads to the same system of equations up to relabelling of nodes. Alternatively, avoiding a mismatch of indices in the terms Φ(u)m, we obtain again that one entry of Φ(u) is out of bounds for each condition. There are two remedies for this problem: either introduce a variable w=Φ(u)m such that the underlying information becomes irrelevant, or introduce fictitious Neumann boundary conditions on *u*, as the fictitious boundary conditions set the four out-of-bounds terms to zero in that case.

We point out that, to the best of our knowledge, neither the convergence of the scheme in [39] nor that of the variant considered in this paper has yet been established. This therefore remains an interesting open problem, which lies outside the scope of the present paper.

### Variational Formulation

As noticed in [3], systems of the form ($\textrm{MFG}_{\mathbb {T}^{d}}$)–($\text {MFG}_{\Omega })$ admit a variational formulation. Define $b:\mathbb {R}\times \mathbb {R}^{d}\rightarrow \mathbb {R}\cup \{+\infty \}$ and, in the periodic case, *F*, $G:\mathbb {T}^{d}\times \mathbb {R}\rightarrow \mathbb {R}\cup \{+\infty \}$ as

$$\begin{aligned} b(m,w)&:= \left\{ \begin{array}{ll} \frac{1}{q}\frac{|w|^{q}}{m^{q-1}} & \text { if } m>0,\\ 0 & \text { if } (m,w)=(0,0), \\ +\infty & \text { otherwise,} \end{array} \right. \\ F(x,m)&:= \left\{ \begin{array}{ll} \int \limits _{0}^{m} f(x,m') \,dm' &  \text{ if } m\ge 0, \\ +\infty &  \text{ otherwise, }\end{array} \right. \\G(x,m)&:= \left\{ \begin{array}{ll} \int \limits _{0}^{m} g(x,m') \,dm' &  \text{ if } m\ge 0, \\ +\infty &  \text{ otherwise, }\end{array} \right. \, \end{aligned}$$

where *q* is the conjugate exponent of $q^{\prime }$, i.e., $q=q^{\prime }/\left(q^{\prime }-1\right)$. System ($$\textrm{MFG}_{\mathbb {T}^{d}}$$) is the optimality system of the variational problem:

**Figure:**
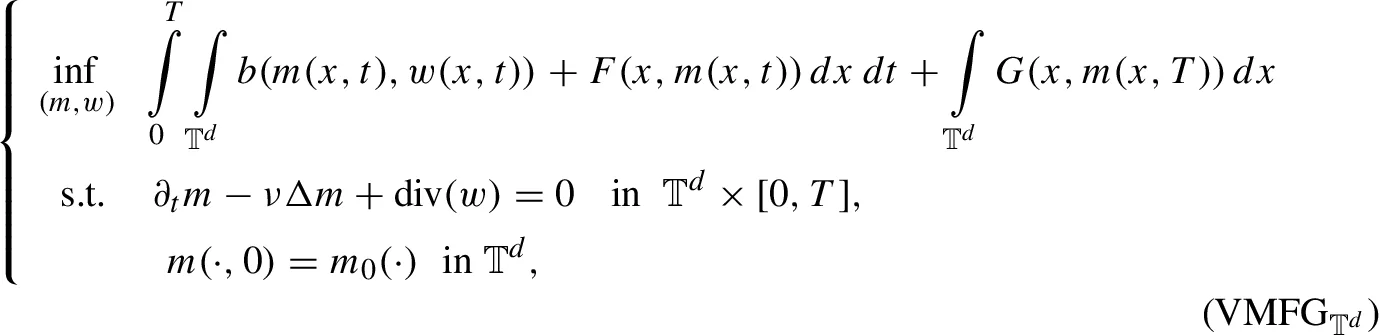

and the system $(\textrm{MFG}_{\Omega })$ is the optimality system of ($\text {VMFG}_{\mathbb {T}^{d}}$) with ${\mathbb {T}^d}$ replaced by Ω and the following additional constraint

$$\begin{aligned} \left( \nu \nabla m-w\right) \cdot n=0 \;\text { on } \;\partial \Omega \times [0,T]. \end{aligned}$$

We denote this variational problem by ($\text {VMFG}_{\Omega })$. Under the assumption that f(x,·) and g(x,·) are non-decreasing, the formulations ($\text {VMFG}_{\mathbb {T}^{d}}$), ($\text {VMFG}_{\Omega })$ are shown to correspond to convex optimization problems and convex duality techniques can be applied to analyse ($\textrm{MFG}_{\mathbb {T}^{d}}$) and ($\text {MFG}_{\Omega })$. See, e.g., [40–43] and the references therein for ($\textrm{MFG}_{\mathbb {T}^{d}}$) or its ergodic version, [44] for a stationary version of ($\text {MFG}_{\Omega })$, and [45] for the first-order case ν=0 in ($\text {MFG}_{\Omega })$.

In the following, we consider the numerical approximations of the continuous optimization problems ($\text {VMFG}_{\mathbb {T}^{d}}$) and ($\text {VMFG}_{\Omega })$, the optimality conditions of which lead to ($\textrm{MFG}_{\mathbb {T}^{2}_{h}}$) and ($\text {MFG}_{\Omega _h})$, respectively. Define $\widehat{b}:\mathbb {R}\times \mathbb {R}^4 \rightarrow \mathbb {R}\cup \{+\infty \}$ as

$$\begin{aligned} \widehat{b}(m,w):= \left\{ \begin{array}{ll} \frac{1}{q}\frac{|w|^{q}}{m^{q-1}}, & \text { if } m>0, ~ w \in \mathcal {K},\\ 0, &  \text { if } (m,w)=(0,0), \\ +\infty , & \text { otherwise}, \end{array} \right. \end{aligned}$$

and for $m:\mathbb {T}^2_h\times T_{\Delta t}\rightarrow \mathbb {R}$ and $w:\mathbb {T}^2_h\times (T_{\Delta t}\setminus \{T\})\rightarrow \mathbb {R}$ set

$$\begin{aligned} \mathcal {B}(m,w)&:=\sum _{i,j,k}\widehat{b}(m_{i,j}^k, w_{i,j}^k), \\ \mathcal {F}(m)&:=\sum _{i,j,k} F(x_{i,j},m_{i,j}^k) + \frac{1}{\Delta t}\sum _{i,j} G(x_{i,j},m_{i,j}^{N_t}), \\ {\mathcal {C}}(m,w)&:=\begin{bmatrix} m_{i,j}^0- m_0(x_{i,j}) \\ \begin{array}{c}(D_tm)^0_{i,j} -\nu (\Delta _hm)^1_{i,j} + (\textrm{div}_h w)^{0}_{i,j} \\ \vdots \\ (D_tm)^{N_t-1}_{i,j} -\nu (\Delta _hm)^{N_t}_{i,j} + (\textrm{div}_h w)^{N_t-1}_{i,j}\end{array} \end{bmatrix}. \end{aligned}$$

Then, problem ($\textrm{MFG}_{\mathbb {T}^{2}_{h}}$) is the optimality condition of the (scaled) discrete variational problem (see, e.g., [21]):

**Figure:**
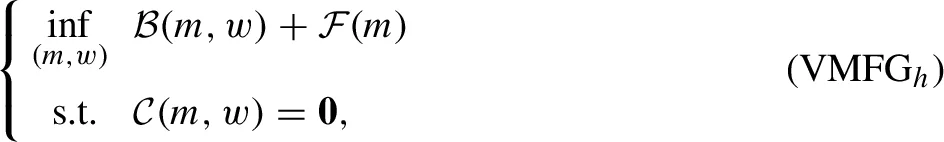

where $\boldsymbol{0}$ stands for a zero vector of $(N_t + 1)N_xN_y$ entries. Applying the initial condition leaves $N_tN_xN_y$ unknowns for the mass and the same number for each component of *w*, leading to a total of $5N_tN_xN_y$ unknowns. Let us mention that the set $\mathcal {K}$ is introduced in the definition of $\widehat{b}$ precisely in order to recover the projection operator $P_{\mathcal {K}}$ in the optimality system ($\textrm{MFG}_{\mathbb {T}^{2}_{h}}$). We refer the reader to [20, 21] for details.

Similarly, problem ($\text {MFG}_{\Omega _h})$ is the optimality condition of the variational problem ($\text {VMFG}_{h}$) with $\mathbb {T}^2_h$ replaced by $\Omega _h$ and the following additional constraints defined on $\partial \Omega _h$:

$$\begin{aligned} -\nu (D_1 m)^{k+1}_{0,j}+(w^{(1)})^k_{0,j} + (w^{(2)})^k_{1,j}&=0, \\ \nu (D_1 m)^{k+1}_{N_x,j}-(w^{(1)})^k_{N_x,j}-(w^{(2)})^k_{N_x+1,j}&=0, \\ -\nu (D_2 m)^{k+1}_{i,0}+(w^{(3)})^k_{i,0}+(w^{(4)})^k_{i,1}&=0, \\ \nu (D_2 m)^{k+1}_{i,N_y}-(w^{(3)})^k_{i,N_y}-(w^{(4)})^k_{i,N_y+1}&=0, \end{aligned}$$

applied at each time step, leading again to a total of $5N_tN_xN_y$ unknowns. Note that the operator $\Delta _h$ is symmetric, hence if we introduce discrete Lagrange multipliers $u^{k}_{0,j},u^{k}_{N_x+1,j},u^{k}_{i,0},u^{k}_{i,N_y+1}$ associated with the Neumann boundary constraints and differentiate the discrete Lagrangian with respect to *m*, we obtain again the discrete Neumann boundary conditions for *u*.

The following sections are devoted to the efficient numerical solution to systems ($\textrm{MFG}_{\mathbb {T}^{2}_{h}}$) and ($\text {MFG}_{\Omega _h})$ through variational and numerical linear algebra techniques.

## A Primal–Dual Method for the Discretized Variational Mean Field Game

In this section we state a version of the primal–dual method introduced in [30] to solve the finite-dimensional convex optimization problem ($\text {VMFG}_{h}$). For a convex optimization problem of the form

$$\begin{aligned} \min _{y\in \mathbb {R}^n}{\varphi (y)+\psi (y)}, \end{aligned}$$

where $\varphi :\mathbb {R}^n\rightarrow \mathbb {R}\cup \{+\infty \}$ and $\psi :\mathbb {R}^n\rightarrow \mathbb {R}\cup \{+\infty \}$ are convex l.s.c. proper functions, the (ℓ+1)-th Chambolle–Pock iteration reads

2 $$\begin{aligned} \begin{aligned} x_{\ell +1}&:= {\textbf{prox}}_{\sigma \psi ^*}(x_{\ell }+ \sigma \tilde{y}_{\ell }),\\ y_{\ell +1}&:= {\textbf{prox}}_{\tau \varphi }(y_{\ell }-\tau x_{\ell +1}),\\ \tilde{y}_{\ell +1}&:= y_{\ell +1}+ \theta (y_{\ell +1}-y_{\ell }), \end{aligned}\end{aligned}$$

where σ>0 and τ>0 satisfy $\sigma \tau <1$, the parameter $\theta \in [0,1]$, $\psi ^*$ denotes the convex conjugate of ψ given by

$$ {\psi ^*:\mathbb {R}^n\mapsto \mathbb {R}\cup \{+\infty \}: x^*\mapsto \;\sup _{x\in \mathbb {R}^n}\left( x^*\cdot x-\psi (x)\right) }, $$

and, for a given a l.s.c. convex proper function $\phi :\mathbb {R}^n\rightarrow \, ]-\infty ,+\infty ]$ and γ>0, the proximal operator of $\gamma \phi$ is defined as

3 $$\begin{aligned} {\textbf{prox}}_{\gamma \phi }x&:= { \underset{y\in \mathbb {R}^n}{\text{ argmin }}\left\{ \gamma \phi (y)+\frac{|y-x|^2}{2}\right\} } \hspace{0.5cm} \forall \; x\in \mathbb {R}^n. \end{aligned}$$

As in [21], we approximate the solution to ($\textrm{MFG}_{\mathbb {T}^{2}_{h}}$) by considering the iterates (2) applied to Problem ($\text {VMFG}_{h}$) with $y=(m,w)^\top$, $\varphi (m,w):=\mathcal {B}(m,w)+\mathcal {F}(m)$, and

$$\begin{aligned} \psi (m,w):={\left\{ \begin{array}{ll} 0 &  \text {if}\;{\mathcal {C}}(m,w)=\boldsymbol{0},\\ +\infty &  \text {otherwise}. \end{array}\right. } \end{aligned}$$

The first and second steps in (2) require the computation of ${\textbf{prox}}_{\sigma \psi ^*}$ and ${\textbf{prox}}_{\tau \varphi }$, respectively. Regarding the latter, it follows directly from (3) that

$$\begin{aligned} \left( {\textbf{prox}}_{\tau \varphi }(m,w)\right) _{i,j}^{k}={\textbf{prox}}_{\tau \varphi _{i,j}}(m_{i,j}^{k},w_{i,j}^k), \end{aligned}$$

where $\varphi _{i,j}(m,w):=\widehat{b}(m,w)+ F(x_{i,j},m)$ for all $(m,w)\in \mathbb {R}\times \mathbb {R}^4$. Therefore, ${\textbf{prox}}_{\tau \varphi }$ can be directly computed from the semi-explicit expressions, involving the solution of a scalar equation, for ${\textbf{prox}}_{\tau \varphi _{i,j}}$ in [20, Corollary 3.1]. For the computation of ${\textbf{prox}}_{\sigma \psi ^*}$, we begin by noting that the constraint ${\mathcal {C}}(m,w)=\boldsymbol{0}$ can be conveniently expressed as the linear system

4 $$\begin{aligned} \textbf{C}(m,w)^\top =\boldsymbol{d}. \end{aligned}$$

Here, $\textbf{C}=[\textbf{A}|\textbf{B}]$, where $\textbf{A}$ is an $(N_t+1)N_xN_y\times (N_t+1)N_xN_y$ matrix arising from the discretization of the term $(\partial _t-\nu \Delta )m$, and $\textbf{B}$ is an $(N_t+1)N_xN_y\times 4(N_t+1)N_xN_y$ matrix arising from the discretization of ${\textrm{div}}(w)$ on $T_{\Delta t}$. The vector $\boldsymbol{d}$ corresponds to the right-hand side of the constraints, which incorporates the initial datum $m_0(x)$. Next, the identity (see, e.g., [46, Section 24.2]) applied to our choice of ψ

$$\begin{aligned} {\textbf{prox}}_{\sigma \psi *}=\textbf{Id}-\sigma {\textbf{prox}}_{\psi /\sigma }\circ (\textbf{Id}/\sigma )=\textbf{Id}-\sigma {\textbf{prox}}_{\psi }\circ (\textbf{Id}/\sigma ), \end{aligned}$$

together with

$$\begin{aligned} {\textbf{prox}}_{\psi }:(m,w)\mapsto (m,w)^\top -\textbf{C}^\top (\textbf{C}\textbf{C}^\top )^{-1}(\textbf{C}(m,w)^\top -{\boldsymbol{d}}) \end{aligned}$$

being determined as the solution of (4) with smallest (squared) 2-norm distance to $(m,w)^\top$, leads to

5 $$\begin{aligned} {\textbf{prox}}_{\sigma \psi *}:(m,w)\mapsto \textbf{C}^\top (\textbf{C}\textbf{C}^\top )^{-1}(\textbf{C}(m,w)^\top -\sigma \boldsymbol{d})\,. \end{aligned}$$

We may now briefly state the structure of the matrices arising from the finite-difference discretization of (4) at each Chambolle–Pock iteration. The matrices $\textbf{A}$ and $\textbf{B}$, arising from the terms $(\partial _t-\nu \Delta )m$ and ${\textrm{div}}(w)$ respectively, are

$$\begin{aligned} \textbf{A}= \left[ \begin{array}{cccccc} I &  0 &  \cdots &  \cdots &  0 &  0 \\ - \frac{1}{\Delta t} I &  L &  0 &  &  &  0 \\ 0 &  - \frac{1}{\Delta t} I &  L &  \ddots &  &  \vdots \\ \vdots &  \ddots &  \ddots &  \ddots &  \ddots &  \vdots \\ 0 &  &  \ddots &  - \frac{1}{\Delta t} I &  L &  0 \\ 0 &  0 &  \cdots &  0 &  - \frac{1}{\Delta t} I &  L \\ \end{array}\right] , \quad \textbf{B}= \left[ \begin{array}{cccccc} 0 &  0 &  \cdots &  \cdots &  0 &  0 \\ B &  0 & &  &  &  0 \\ 0 &  B &  \ddots & &  &  \vdots \\ \vdots &  &  \ddots &  \ddots &  &  \vdots \\ 0 &  &  &  B &  0 &  0 \\ 0 &  0 &  \cdots &  0 &  B &  0 \\ \end{array}\right] . \end{aligned}$$

Here, *I* is the identity matrix, *B* discretizes the divergence operator at each time step, and $L = \nu K + \frac{1}{\Delta t} I$ with *K* discretizing the negative Laplacian operator. The matrices *L* and *I* are symmetric positive definite, with *K* and $B B^\top$ symmetric positive semidefinite.

Upon forming $\textbf{C}\textbf{C}^\top$, which is equal to $\textbf{A}\textbf{A}^\top +\textbf{B}\textbf{B}^\top$ in the notation above, we wish to solve a system involving $\textbf{C}\textbf{C}^\top$, as required for (5). We may eliminate the first ($N_xN_y \times N_xN_y$) block row, arising from the initial condition, and are then left with a reduced $N_tN_xN_y\times N_tN_xN_y$ system of the form $\boldsymbol{\mathcal {A}}\boldsymbol{x}=\boldsymbol{b}$, where

6 $$\begin{aligned} \boldsymbol{\mathcal {A}} = \left[ \begin{array}{cccccc} C &  - \frac{1}{\Delta t} L &  0 &  \cdots &  0 &  0 \\ - \frac{1}{\Delta t} L &  C + \frac{1}{(\Delta t)^2} I &  - \frac{1}{\Delta t} L &  \ddots &  &  0 \\ 0 &  - \frac{1}{\Delta t} L &  C + \frac{1}{(\Delta t)^2} I &  - \frac{1}{\Delta t} L &  &  \vdots \\ \vdots &  \ddots &  \ddots &  \ddots &  \ddots &  0 \\ 0 &  &  \ddots &  - \frac{1}{\Delta t} L &  C + \frac{1}{(\Delta t)^2} I &  - \frac{1}{\Delta t} L \\ 0 &  0 &  \cdots &  0 &  - \frac{1}{\Delta t} L &  C + \frac{1}{(\Delta t)^2} I \\ \end{array}\right] , \end{aligned}$$

where $C = L^2 + B B^\top$. As $\boldsymbol{\mathcal {A}}$ can be a huge-scale matrix, particularly upon fine discretizations of the space and time variables, it is particularly important to be able to accurately and efficiently solve linear systems of this form. In the next section, we therefore consider how to effectively precondition the matrix $\boldsymbol{\mathcal {A}}$, with the goal that such a preconditioner may be applied within the conjugate gradient (CG) algorithm [47].

## Parallel-in-Time Iterative Solver

Depending on the algebraic properties of the matrix $\boldsymbol{\mathcal {A}}$ from (6) that arises at each Chambolle–Pock (CP) iteration, the inverse can be directly computed, or the system can be solved directly. However, both options are expensive computationally and storing the potentially dense decompositions required for an exact solve is costly. Instead, the resulting system can be solved using a preconditioned Krylov subspace method. Note that the matrix $\boldsymbol{\mathcal {A}}$ from (6) is symmetric positive definite, hence we can use the CG method [47]. However, as the system arising at each CP iteration is of very large scale, and is expected to be ill-conditioned with a fine mesh, preconditioning, i.e., transforming the system into a convenient form for the chosen solver, becomes essential. A preconditioner used within CG will also need to be symmetric positive definite.

A limited number of preconditioning strategies for the system (6) have been explored in the literature, most popularly multigrid methods and incomplete Cholesky factorization as in [21], which both show satisfactory performance, particularly for large ν. For systems similar to (6), multigrid methods [34] and a block-diagonalizable preconditioner using a temporal transformation and multigrid in space [19] have been applied. However, the former method, although developed on the discrete level, does not involve the operator $BB^\top$, and the latter is a continuous operator preconditioning approach involving a system $\textbf{C}^{\top }\textbf{C}$ resembling many of the above structures and a temporal discretization similar to continuous Galerkin time-stepping. We employ a “discretize-in-space-first” approach and deal with the exact matrices that arise. Two further approaches have been explored during this study: (i) a cheap-to-apply preconditioner neglecting the matrix $BB^\top$ where Chebyshev semi-iteration is used to approximate the inverse action of *L*; (ii) a refined preconditioner tailored to the regime of a small viscosity ν, employing a matching strategy [48, 49] and an incomplete Cholesky decomposition of the positive semidefinite matrix $BB^\top$. Given its triangular structure, the terms within the decomposition of $BB^\top$ are then applied using Gauss–Seidel iteration within the matching strategy. Both preconditioners show satisfactory performance, with the second showing generally superior performance in numerical tests as it incorporates more information about the underlying system, and is more robust with respect to the parameter ν. In this paper, we instead derive suitable preconditioners which can be applied in parallel for each time step by employing real-valued DFTs to block-diagonalize the huge-scale system in the time variable (often referred to as a ParaDiag approach), which we find to be a superior approach in practice.

The derivation of parallel-in-time methods for PDEs is an area of increasing interest within numerical mathematics; we refer to [50, 51] for surveys. Alongside approaches such as the parareal scheme [52, 53], other multiple shooting strategies [54–56], and space–time multigrid approaches like Parallel Full Approximation Scheme in Space and Time (PFASST) [57, 58] and multigrid reduction-in-time (MGRIT) [59, 60], all of which may be analysed in a unified manner [61], an emerging and active area of research is to treat the resulting linear systems within the ParaDiag fashion [62–64]. We employ this philosophy within the present work, as this enables the resolution of highly-challenging systems via fast trigonometric transforms and a block diagonal solver as described above.

Parallel-in-time preconditioning approaches frequently arise from recognizing underlying symmetries and repeating block structures, a well-established principle in preconditioning [65]. To this end, note that the matrix $\boldsymbol{\mathcal {A}}$ has a uniform pattern of diagonal and off-diagonal matrices, apart from the first $N_xN_y\times N_xN_y$ block. Assuming suitable boundary, initial, and possibly final conditions, the matrix $\boldsymbol{\mathcal {A}}$ can be shown to be similar to a recursive linear algebra decomposition involving a combination of negative Laplacian operators. In the zero viscosity case, the system $\boldsymbol{\mathcal {A}}\boldsymbol{x}=\boldsymbol{b}$ is similar to the system arising from a three-dimensional Poisson equation, which can be solved exactly using discrete cosine transforms (DCTs), as noticed by Papadakis et al. [35]. Conveniently, the discrete negative Laplacian operator can be diagonalized by a decomposition of the form $V^{-1} \Lambda V$, where *V* is a DFT matrix (or a linear combination of DFTs in the form of DCTs or discrete sine transforms (DSTs)) determined by suitable boundary and initial (and final) conditions, and Λ is a diagonal matrix containing the eigenvalues of the negative Laplacian operator. We extend the solution strategy presented in [35] to the full MFG with non-zero viscosity by constructing suitable positive definite preconditioners on the discrete level for periodic and Neumann boundary conditions. Nevertheless, we allow for other geometries and non-constant viscosity ν(x) when applying the parallel-in-time preconditioning technique, as it remains true that the matrices discretizing the spatial derivatives are the same at every time step. We omit the final condition included in the optimal transport problem [35] (which would otherwise lead to the MFG planning problem [25]) and treat any target state as a penalty term in the objective function.

The main advantage of this approach is the parallelization capability of the preconditioners. The system arising at each time step can be solved in parallel with a number of solution methods, including LU factorization, AMG, or by extending the recursive linear algebra technique to also include spatial decompositions. The following sections describe two options for the decomposition in time, a special no-viscosity case, and lastly two possible spatial decompositions covering the periodic and Neumann boundary condition settings, in the case of a rectangular grid.

### Block-Diagonalizable Preconditioner in Time

We start by devising a preconditioned approximation for the discretization in time. Recall that $L=\nu K + \frac{1}{\Delta t}I_{N_xN_y}$, where from now on the subscript of an identity matrix corresponds to its dimension. Hence, on the discrete level we have

$$\begin{aligned} C=L^2 + BB^\top =\hat{C} + \frac{2\nu }{\Delta t} K + \frac{1}{(\Delta t)^2}I_{N_xN_y}, \end{aligned}$$

where

$$\begin{aligned} \hat{C}=\nu ^2 K^2 + BB^\top . \end{aligned}$$

Letting $H=\frac{1}{\Delta t} (2\nu K + \frac{1}{\Delta t}I_{N_xN_y})$ and $\hat{L} = \frac{1}{\Delta t}L$, then $\boldsymbol{\mathcal {A}}$ in (6) is of the form

7 $$\begin{aligned} \boldsymbol{\mathcal {A}} = \begin{bmatrix} \hat{C} + H &  - \hat{L} &  0 &  \cdots &  0 &  0 \\ - \hat{L} &  \hat{C} + 2\hat{L} &  - \hat{L} &  \ddots &  &  0 \\ 0 &  -\hat{L} &  \hat{C} + 2\hat{L} &  -\hat{L} &  \ddots &  \vdots \\ \vdots &  \ddots &  \ddots &  \ddots &  \ddots &  0 \\ 0 &  &  \ddots &  - \hat{L} &  \hat{C} + 2\hat{L} &  -\hat{L} \\ 0 &  0 &  \cdots &  0 &  -\hat{L} &  \hat{C} + 2\hat{L} \end{bmatrix}. \end{aligned}$$

Note that we can approximate *H* with a scalar multiple of $\hat{L}$ by either subtracting a term $\frac{\nu }{\Delta t}K$, or adding a term $\frac{1}{(\Delta t)^2} I_{N_xN_y}$, in the first diagonal block, corresponding to setting l=1 and l=2 in $H\approx l\hat{L}$, respectively. Equivalently, consider a preconditioner of the form

8 $$\begin{aligned} \boldsymbol{\mathcal {P}} = \begin{bmatrix} \hat{C} + l \hat{L} &  - \hat{L} &  0 &  \cdots &  0 &  0 \\ - \hat{L} &  \hat{C} + 2\hat{L} &  - \hat{L} &  \ddots &  &  0 \\ 0 &  -\hat{L} &  \hat{C} + 2\hat{L} &  -\hat{L} &  \ddots &  \vdots \\ \vdots &  \ddots &  \ddots &  \ddots &  \ddots &  0 \\ 0 &  &  \ddots &  - \hat{L} &  \hat{C} + 2\hat{L} &  -\hat{L} \\ 0 &  0 &  \cdots &  0 &  -\hat{L} &  \hat{C} + 2\hat{L} \end{bmatrix}. \end{aligned}$$

We present the following lemma for the spectral properties of the preconditioned matrix $\boldsymbol{\mathcal {P}}^{-1}\boldsymbol{\mathcal {A}}$, which provide a guarantee for the convergence properties of the preconditioned system in an ideal setting.

#### Lemma 1

Suppose $\boldsymbol{\mathcal {A}}$ and $\boldsymbol{\mathcal {P}}$ are defined as in (7) and (8), respectively, with two spatial dimensions $N_x$, $N_y$. Then 1 is an eigenvalue of $\boldsymbol{\mathcal {P}}^{-1}\boldsymbol{\mathcal {A}}$ with algebraic multiplicity at least $(N_t-1)N_xN_y$, and the remaining $N_xN_y$ eigenvalues are of the form 1+μ, with μ being eigenvalues of the matrix $\boldsymbol{\mathcal {P}}^{-1}\boldsymbol{\mathcal {H}},$ where

$$\begin{aligned} \boldsymbol{\mathcal {H}} = \begin{bmatrix} H - l\hat{L} &  0 &  \cdots &  0 \\ 0 &  0 &  \ddots &  \vdots \\ \vdots &  \ddots &  \ddots &  0 \\ 0 &  \cdots &  0 &  0 \end{bmatrix}. \end{aligned}$$

#### Proof

Consider the eigenvalue problem

9 $$\begin{aligned} \boldsymbol{\mathcal {A}}\boldsymbol{x}=\lambda \boldsymbol{\mathcal {P}}\boldsymbol{x}, \end{aligned}$$

where $\boldsymbol{x}$ is the eigenvector associated with eigenvalue λ. Note that $\boldsymbol{\mathcal {A}} = \boldsymbol{\mathcal {P}} + \boldsymbol{\mathcal {H}}$. Hence the eigenvalue problem (9) becomes

10 $$\begin{aligned} \boldsymbol{\mathcal {P}}\boldsymbol{x} + \boldsymbol{\mathcal {H}}\boldsymbol{x}=\lambda \boldsymbol{\mathcal {P}}\boldsymbol{x}\phantom {\mu } \quad \iff \quad \phantom {\mu }(\lambda - 1)\boldsymbol{\mathcal {P}}\boldsymbol{x}&= \boldsymbol{\mathcal {H}} \boldsymbol{x} \nonumber \\ \iff \quad \phantom {\boldsymbol{\mathcal {P}}(\lambda - 1)}\mu \boldsymbol{x}&= \boldsymbol{\mathcal {P}}^{-1}\boldsymbol{\mathcal {H}} \boldsymbol{x}, \end{aligned}$$

where $\mu =\lambda -1$. Note that by the dimension of the problem that there are at most $N_tN_xN_y$ linearly independent non-trivial solutions $\boldsymbol{x}$ to the eigenvalue problem (10). The matrix $\boldsymbol{\mathcal {H}}$ is made up of mostly zeros and has rank at most $N_xN_y$. Hence there are at least $(N_t-1)N_xN_y$ linearly independent solutions $\boldsymbol{x}$ which belong to the nullspace of the matrix in (10). For these $(N_t-1)N_xN_y$ solutions, the eigenvalue problem (10) reduces to

$$\begin{aligned} (\lambda -1)\boldsymbol{x}={\boldsymbol{0}}, \end{aligned}$$

which has the unique solution λ=1. Therefore, the preconditioned matrix $\boldsymbol{\mathcal {P}}^{-1}\boldsymbol{\mathcal {A}}$ has at least $(N_t-1)N_xN_y$ eigenvalues equal to 1, and the remaining $N_xN_y$ are of the form $\lambda =1+\mu$, where μ is a solution to (10). □

**Fig. 1:**
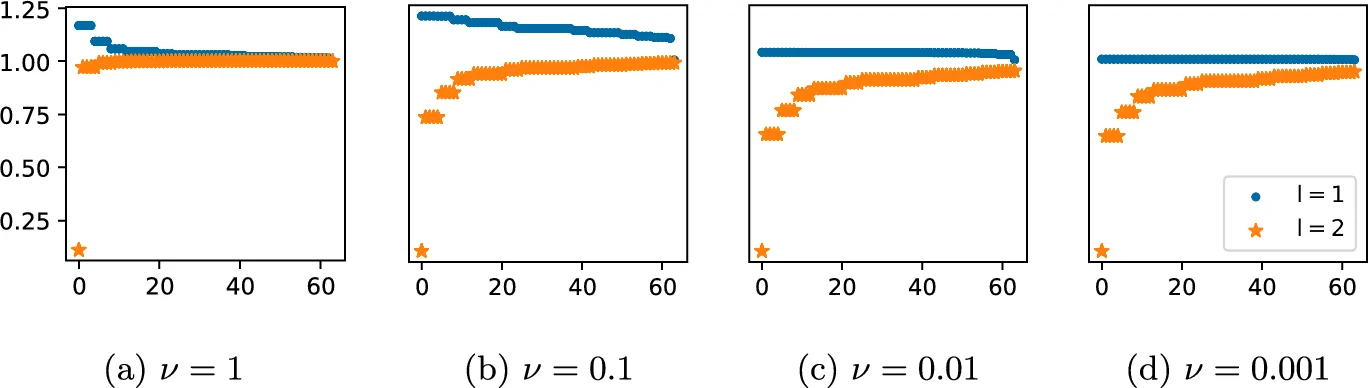
Non-unity eigenvalues of $\boldsymbol{\mathcal {P}}^{-1}{\boldsymbol{\mathcal {A}}}$ for different viscosity parameters ν, and $N_x=N_y=N_t=8$. The horizontal axis contains the labels of the $N_xN_y$ eigenvalues, and the vertical axis displays the eigenvalues

Figure 1 provides a quantitative comparison of the choice $l\in \{1,2\}$, showing the $N_xN_y$ eigenvalues of $\boldsymbol{\mathcal {P}}^{-1}\boldsymbol{\mathcal {A}}$ which may not be equal to 1, for varying viscosity ν. For l=2, one eigenvalue is relatively small in magnitude compared to the rest. However the eigenvalues remain bounded and consistent for all viscosity parameter values. For l=1, the spectrum of eigenvalues is small, with more non-unity eigenvalues in the particular case ν=0.1. As the viscosity decreases, the eigenvalues converge to 1. This pattern can be shown to be consistent also in larger problem instances.

The preconditioner (8) can be decomposed into the following sum of Kronecker products:

$$\begin{aligned} \boldsymbol{\mathcal {P}}&= I_{N_t} \otimes \hat{C} + D_{tt} \otimes \hat{L}, \end{aligned}$$

where $D_{tt}$ is a (scaled) negative second-order finite-difference operator

$$\begin{aligned} D_{tt} = \begin{bmatrix} l &  -1 &  0 &  \cdots &  0 \\ -1 &  2 &  -1 &  \ddots &  \vdots \\ 0 &  -1 &  2 &  \ddots &  0 \\ \vdots &  \ddots &  \ddots &  \ddots &  -1 \\ 0 &  \cdots &  0 &  -1 &  2 \end{bmatrix} \in \mathbb {R}^{{N_t} \times {N_t}}. \end{aligned}$$

Such an operator can be diagonalized by a decomposition of the type $D_{tt} = V^{-1} \Lambda _t V$, where *V* is a matrix transform, either a type VIII DST [66] (l=1) or a type I DST [67] (l=2), and $\Lambda _t$ is a diagonal matrix containing the eigenvalues of $D_{tt}$:

11 $$\begin{aligned} \lambda _k = -2\cos \left( \frac{\pi (k-1+\frac{l}{2})}{N_t+\frac{l}{2}}\right) +2, \quad k = 1, ..., N_t. \end{aligned}$$

The preconditioner can therefore be written as

12 $$\begin{aligned} \boldsymbol{\mathcal {P}}&= I_{N_t} \otimes \hat{C} + (V^{-1} \Lambda _t V) \otimes \hat{L} \nonumber \\&= (V^{-1} \otimes I_{N_xN_y})(I_{N_t} \otimes \hat{C} + \Lambda _t \otimes \hat{L})(V \otimes I_{N_xN_y}). \end{aligned}$$

Note that changing from one preconditioner to the other (l=1, l=2) requires only that the chosen transformation *V* corresponds to the correct eigenvalues $\lambda _k$ in (11). The parameter *l* allows the closest approximation of (6) to a preconditioner which permits a trigonometric/DFT decomposition. We could additionally alter, e.g., the last diagonal block in order to obtain a DCT-II or IV decomposition, but this could require more computational work and would be expected to lead to worse results than the simpler choices of l=1 or l=2. We also note that for the system $\textbf{C}^{\top }\textbf{C}$ arising in ADMM, a DST-II or VII would be the most straightforward choice.

The inversion of the preconditioner (12) is described in Algorithm 1 in terms of its inverse action on an input vector $\boldsymbol{y}$. We first reshape the input vector such that the matrix transformation *V* can be applied to each column vector of dimension $N_t$, i.e., one application of *V* is applied to the vector with precisely one corresponding entry from each of the subvectors $\underline{y}_k$, $k=1,...,N_t$. The preconditioner (12) allows a parallel-in-time implementation when solving the system

13 $$\begin{aligned} (\hat{C} + \lambda _k \hat{L}) \underline{x}_k = \underline{y}_k \end{aligned}$$

for the inverse action of $\hat{C} + \lambda _k \hat{L}$ applied to an input vector $\underline{y}_k$ at each time step *k*. In the last step of the procedure in Algorithm 1, the inverse transformation of *V* is applied in the corresponding dimension, and the matrix solution reshaped into a column vector.

**Algorithm 1:**
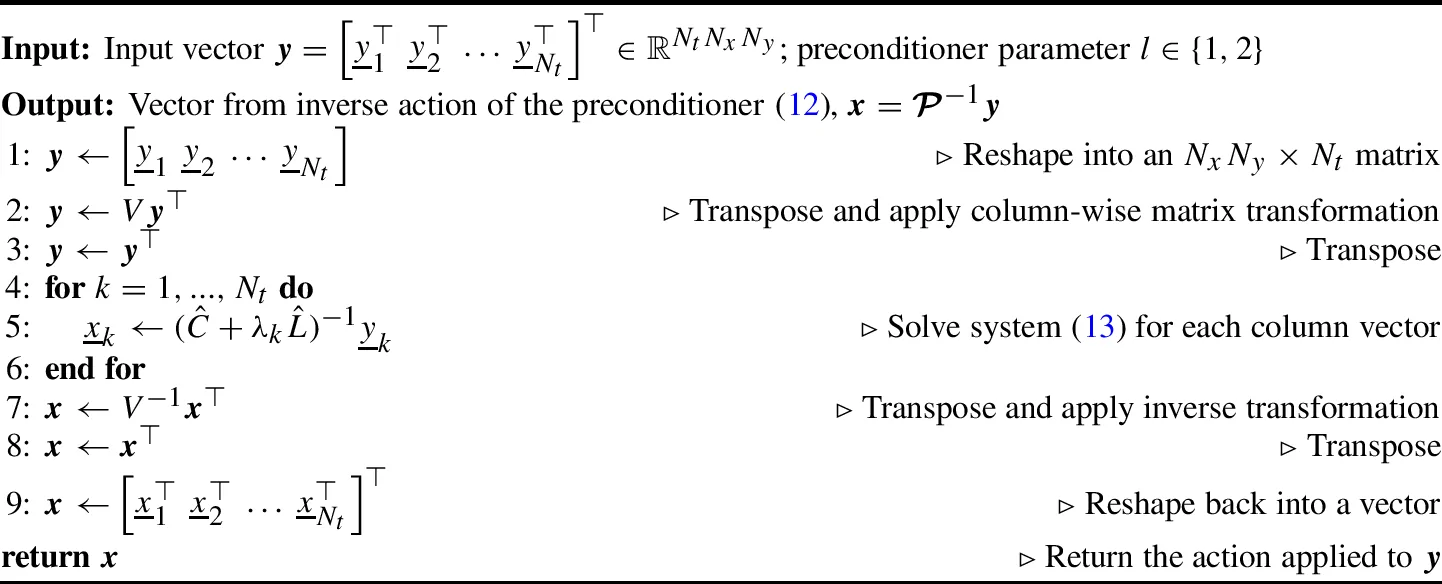
Application of the inverse action of $\boldsymbol{\mathcal {P}}$ in (12)

When solving the system (13), there are a few options to consider in order to reduce runtime and memory. Firstly, the system can be solved exactly, using, e.g., an LU decomposition. This is a convenient method which is readily available with most commercial software. However, the runtime may be excessive for large problems. An incomplete Cholesky decomposition or an AMG method can be applied to reduce memory usage and runtime, but with the caveat that one obtains an approximate rather than exact solution. In the case of a rectangular grid and constant viscosity, we can directly exploit the internal structure of the finite-difference operators and consider a DFT or DCT decomposition in space. We describe this approach in Sect. 4.3. Note also that in the no-viscosity case, the variational MFG with $q^{\prime }=2$ and f≡0 reduces to the Benamou–Brenier formulation of optimal transport without a final constraint [68], in which case the DCT-VIII decomposition in time, corresponding to the preconditioner (12) with l=1, is exact. We explain briefly in the following subsection this special case.

### The Zero Viscosity Case

For small viscosity, the discrepancy between *H* and $\hat{L}$ in the first diagonal block in $\boldsymbol{\mathcal {A}}$ (7) vanishes, since

$$\begin{aligned} \lim _{\nu \rightarrow 0} H = \frac{1}{(\Delta t)^2} I_{N_xN_y} = \lim _{\nu \rightarrow 0} \hat{L}. \end{aligned}$$

Therefore, $\boldsymbol{\mathcal {P}}$ with l=1 approaches $\boldsymbol{\mathcal {A}}$ as we decrease the viscosity, as subtracting the term involving νK makes little to no difference. For small viscosities $0<\nu \ll 1$, we therefore expect this preconditioner to perform well. For ν=0, an exact solution method can be employed. As observed in [35], the divergence operator $\text {div}$ applied to its adjoint operator, defined in terms of the $L^2$ inner product, is closely related to the negative Laplacian operator -Δ, with some additional boundary terms. Also, the first order derivative $\partial _t$ with respect to time applied to its adjoint is closely related to the one-dimensional negative Laplacian operator -Δ. Solving the system $\boldsymbol{\mathcal {\hat{A}}}\boldsymbol{z} = \boldsymbol{b}$ without viscosity is therefore related to solving, on the continuous level, a three-dimensional Poisson equation:

$$\begin{aligned} -\Delta z = b. \end{aligned}$$

Let $D_{tt}$, $D_{xx}$, $D_{yy}$ denote the (scaled) discrete negative Laplacian (or second derivative) operators. The discrete three-dimensional Laplacian operator $\boldsymbol{\mathcal {\hat{A}}}$ can be decomposed into the following sum of Kronecker products:

$$\begin{aligned} \boldsymbol{\mathcal {\hat{A}}}&= \frac{1}{(\Delta t)^2}D_{tt} \otimes I_{N_x} \otimes I_{N_y} + \frac{1}{(\Delta x)^2}I_{N_t} \otimes D_{xx} \otimes I_{N_y} + \frac{1}{(\Delta y)^2}I_{N_t} \otimes I_{N_x} \otimes D_{yy}, \end{aligned}$$

allowing an exact solution using the DFT [69] or a real-valued modification thereof in the form of DSTs/DCTs. In [35], both an initial and a final condition are assumed, leading to

$$\begin{aligned} D_{tt} = \begin{bmatrix} 1 &  -1 &  0 &  \cdots &  0 \\ -1 &  2 &  -1 &  \ddots &  \vdots \\ 0 &  -1 &  \ddots &  \ddots &  0 \\ \vdots &  \ddots &  \ddots &  2 &  -1 \\ 0 &  \cdots &  0 &  -1 &  1 \end{bmatrix} \in \mathbb {R}^{{N_t} \times {N_t}}, \end{aligned}$$

with Neumann boundary conditions in space. Including also a final condition, the problem can be easily solved with three-dimensional DCT-II decomposition, which is a readily available solver in most common computing languages. We consider only an initial condition and include the option to apply a final penalty for any final misfit. Without a final condition, the lesser known DCT-VIII transformation can be used, however it is not available within popular scientific computing packages and requires a bespoke implementation.

When extending the idea of recursive solution methods from optimal transport problems to variational MFGs with non-zero viscosity, no direct solution method exists as the system is no longer similar to a Poisson equation. Rather, we obtain a fourth-order operator $KK^\top$ appearing in each diagonal block $\hat{C}$, which is notoriously ill-conditioned [34]. Therefore, the system will need to be brought into a form that is easily solvable numerically. We describe in the following section how we construct suitable decompositions in space for the discrete operators in $\boldsymbol{\mathcal {A}}$, arising from a “discretize-in-space-first” approach, for periodic and Neumann boundary conditions on a rectangular grid and $\nu \ge 0$.

### Recursive Preconditioner

We describe in this section the internal structure of the matrices *K* and *B* arising in $\hat{C}$ and $\hat{L}$ in (7), leading to a set of fully recursive preconditioners in both time and space. Turning first to the divergence operator *B*, we treat the momentum variable *w* separately in terms of its positive and negative components, as done in [20, 21]. The discrete divergence operator *B* in one spatial dimension is of the form $\frac{1}{\Delta x}[D^1_x, D^2_x]$, where $D^1_x,D_x^2$ are (scaled) finite-difference divergence operators. For periodic boundary conditions, we have $D^2_x=-(D^1_x)^\top$, where $D^1_x$ is defined as

$$ \begin{bmatrix} 1 &  0 &  \cdots &  0 &  -1 \\ -1 &  1 &  0 &  &  0 \\ 0 &  -1 &  \ddots &  \ddots &  \vdots \\ \vdots &  \ddots &  \ddots &  1 &  0 \\ 0 &  \cdots &  0 &  -1 &  1 \end{bmatrix}. $$

For the Neumann problem, the operators $D^1_x$, $D_x^2$ are (scaled) finite-difference divergence operators with Dirichlet-type boundary conditions, respectively:

$$ \begin{bmatrix} 1 &  0 &  0 &  \cdots &  0 \\ -1 &  1 &  0 &  &  \vdots \\ 0 &  -1 &  \ddots &  \ddots &  0 \\ \vdots &  \ddots &  \ddots &  1 &  0 \\ 0 &  \cdots &  0 &  -1 &  0 \end{bmatrix}, \quad \begin{bmatrix} 0 &  1 &  0 &  \cdots &  0 \\ 0 &  -1 &  1 &  \ddots &  \vdots \\ 0 &  0 &  \ddots &  \ddots &  0 \\ \vdots &  &  \ddots &  -1 &  1 \\ 0 &  \cdots &  0 &  0 &  -1 \end{bmatrix}. $$

Note that all the matrices are of size $N_x\times N_x$, while $\Delta x = (b-a)/N_x$ in the periodic case and $\Delta x = (b-a)/(N_x+1)$ in the Neumann case. Now, the discrete divergence operators multiplied with their transpose, $D^1_x (D^1_x)^\top$ and $D^2_x (D^2_x)^\top$, in the periodic and Neumann boundary condition settings respectively, are both equal to

$$ \begin{bmatrix} 2 &  -1 &  0 &  \cdots &  0 &  -1 \\ -1 &  2 &  -1 &  \ddots &  &  0 \\ 0 &  -1 &  2 &  \ddots &  \ddots &  \vdots \\ \vdots &  \ddots &  \ddots &  \ddots &  -1 &  0 \\ 0 &  &  \ddots &  -1 &  2 &  -1 \\ -1 &  0 &  \cdots &  0 &  -1 &  2 \end{bmatrix}, \quad \begin{bmatrix} 1 &  -1 &  0 &  \cdots &  0 &  0 \\ -1 &  2 &  -1 &  \ddots &  &  0 \\ 0 &  -1 &  2 &  \ddots &  \ddots &  \vdots \\ \vdots &  \ddots &  \ddots &  \ddots &  -1 &  0 \\ 0 &  &  \ddots &  -1 &  2 &  -1 \\ 0 &  0 &  \cdots &  0 &  -1 &  1 \end{bmatrix}. $$

Adding these we have, after scaling, $BB^\top = \frac{1}{(\Delta x)^2}(D^1_x (D^1_x)^\top + D^2_x (D^2_x)^\top )$, or, respectively,

$$ \frac{2}{(\Delta x)^2}\begin{bmatrix} 2 &  -1 &  0 &  \cdots &  0 &  -1 \\ -1 &  2 &  -1 &  \ddots &  &  0 \\ 0 &  -1 &  2 &  \ddots &  \ddots &  \vdots \\ \vdots &  \ddots &  \ddots &  \ddots &  -1 &  0 \\ 0 &  &  \ddots &  -1 &  2 &  -1 \\ -1 &  0 &  \cdots &  0 &  -1 &  2 \end{bmatrix}, \quad \frac{2}{(\Delta x)^2}\begin{bmatrix} 1 &  -1 &  0 &  \cdots &  0 &  0 \\ -1 &  2 &  -1 &  \ddots &  &  0 \\ 0 &  -1 &  2 &  \ddots &  \ddots &  \vdots \\ \vdots &  \ddots &  \ddots &  \ddots &  -1 &  0 \\ 0 &  &  \ddots &  -1 &  2 &  -1 \\ 0 &  0 &  \cdots &  0 &  -1 &  1 \end{bmatrix}. $$

For this discretization, the discrete divergence operator multiplied with its transpose, $BB^\top$, is precisely two times the negative Laplacian operator with periodic or Neumann boundary conditions, i.e., $BB^\top =2K$. Hence we have

14 $$\begin{aligned} \hat{C}=\nu ^2 K^2 + 2K. \end{aligned}$$

The discrete Laplacian operator with periodic boundary conditions is circulant and permits a DFT decomposition, and with Neumann boundary conditions the matrix permits a DCT-II decomposition. Assume the discrete negative Laplacian operator *K* permits one of the two decompositions, $K=\frac{1}{(\Delta x)^2}H^{-1}\Lambda _x H$, scaled by one spatial dimension, and with $\Lambda _x$ containing the eigenvalues of the negative Laplacian matrix. Since the Laplacian is symmetric, squaring this operator is equivalent to squaring only the diagonal matrix in the respective decomposition:

$$\begin{aligned} K^2&= \frac{1}{(\Delta x)^4}(H^{-1}\Lambda _x H)(H^{-1}\Lambda _x H) =\frac{1}{(\Delta x)^4}H^{-1}\Lambda _x^2 H. \end{aligned}$$

Therefore, the matrix $\hat{C}$ in (14) can be expressed in the convenient form

$$\begin{aligned} \hat{C}= \frac{\nu ^2}{(\Delta x)^4} H^{-1}\Lambda _x^2\,H + \frac{2}{(\Delta x)^2}H^{-1}\Lambda _x H=H^{-1}\left( \frac{\nu ^2}{(\Delta x)^4}\Lambda _x^2+\frac{2}{(\Delta x)^2}\Lambda _x\right) H, \end{aligned}$$

Note that this provides an easy extension from the optimal transport problem to the more general variational MFGs, as we can set $\nu \ge 0$ and retain the same decomposition.

Considering now two spatial dimensions, suppose $D_{xx}=H^{-1}\Lambda _xH$ and $D_{yy}=G^{-1}\Lambda _yG$, then the negative Laplacian operator is

$$\begin{aligned} K&=\frac{1}{(\Delta x)^2}D_{xx}\otimes I_y + \frac{1}{(\Delta y)^2}I_x \otimes D_{yy} \\&= (I_{N_x}\otimes G^{-1})(H^{-1} \otimes I_{N_y})D (H \otimes I_{N_y})(I_{N_x}\otimes G), \end{aligned}$$

where

$$\begin{aligned} D = \frac{1}{(\Delta x)^2}\Lambda _x\otimes I_{N_y} + \frac{1}{(\Delta y)^2}I_{N_x}\otimes \Lambda _y. \end{aligned}$$

The matrix (14) can therefore be simply decomposed as follows:

$$\begin{aligned} \hat{C} = (I_{N_x}\otimes G^{-1})(H^{-1} \otimes I_{N_y})\Lambda _1 (H \otimes I_{N_y})(I_{N_x}\otimes G), \end{aligned}$$

where

15 $$\begin{aligned} \Lambda _1&= \nu ^2 D^2 + 2D. \end{aligned}$$

Similarly, we have

$$\begin{aligned} \hat{L}&= \frac{1}{\Delta t}\left( \nu K + \frac{1}{\Delta t}I_{N_xN_y}\right) \\&= (I_{N_x}\otimes G^{-1})(H^{-1} \otimes I_{N_y})\Lambda _2(H \otimes I_{N_y})(I_{N_x}\otimes G), \end{aligned}$$

where

16 $$\begin{aligned} \Lambda _2&= \frac{1}{\Delta t}\left( \nu D + \frac{1}{\Delta t}I_{N_x} \otimes I_{N_y}\right) . \end{aligned}$$

Hence the matrix (13) arising at each time step *k* is

17 $$\begin{aligned} \hat{C} + \lambda _k \hat{L} = (I_{N_x}\otimes G^{-1})(H^{-1} \otimes I_{N_y})(\Lambda _1 + \lambda _k \Lambda _2) (H \otimes I_{N_y})(I_{N_x}\otimes G). \end{aligned}$$

This is a combination of matrix transforms and diagonal matrices, allowing an easy inversion. The pseudocode for inverting the matrix (17) is given in Algorithm 2. First, we define the eigenvalues of the negative Laplacian. Although the Laplacian with periodic or Neumann boundary conditions has a zero eigenvalue, a perturbation parameter is not needed in the time-dependent case due to the presence of $\frac{1}{(\Delta t)^2}I_{N_xN_y}$ in $\hat{L}$. Note also that the implementation allows mixed boundary conditions in the *x* and *y* directions, and extends easily to higher dimensions. The notation $$\begin{bmatrix} {\overline{y}}^\top _1&\overline{y}^\top _2&\ldots&\overline{y}^\top _{N_x}\end{bmatrix}^\top \in \mathbb {R}^{N_xN_y}$$, refers to $N_x$ subvectors each of length $N_y$, i.e., $\overline{y}_i$ contains information about $\underline{y}$ at all spatial nodes in the second axis for index *i* in the first axis. The diagonal matrix in (17) can be reshaped into a convenient form *S*, leading to a computationally cheap entry-wise inversion on line 14. Line 10 refers to the addition of a constant to all entries of *S*. After reshaping $\underline{y}$ accordingly, a DFT or DCT is applied depending on the boundary conditions, along the *y*-direction first (line 12), and then the *x*-direction (line 13). After dividing by the eigenvalues, the inverse transformation is applied in order on lines 15–16. Lastly, the resulting matrix is reshaped into a vector.

**Algorithm 2:**
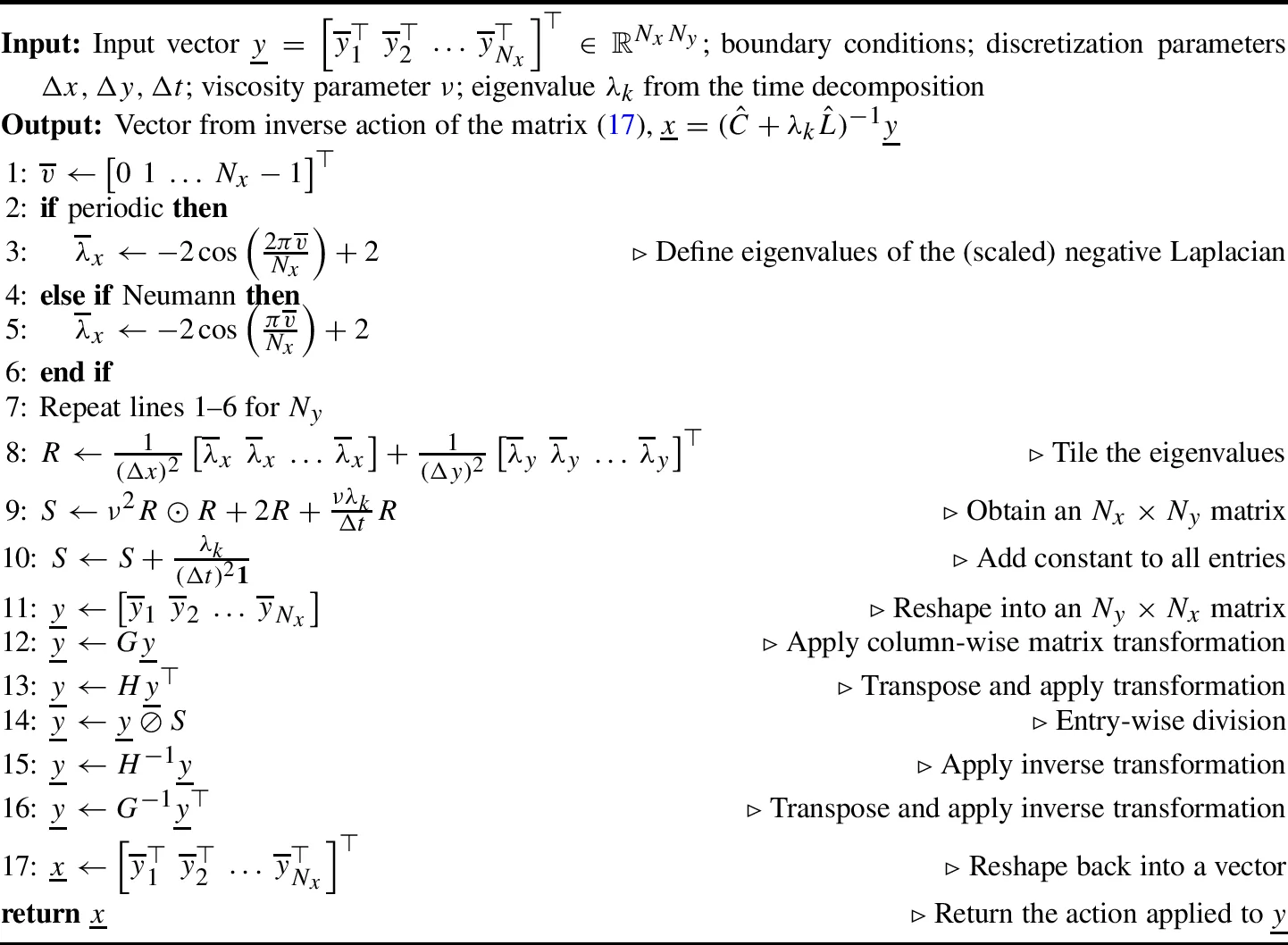
Application of the inverse action of $\hat{C}+\lambda _k\hat{L}$ in (17)

Adding the decomposition in space to the time-dependent problem corresponds to adding layers to the recursive preconditioner (12), that is

18 $$\begin{aligned} \boldsymbol{\mathcal {P}} = (V^{-1} \otimes H^{-1} \otimes G^{-1}) (I_{N_t} \otimes \Lambda _1 + \Lambda _t \otimes \Lambda _2)(V \otimes H\otimes G), \end{aligned}$$

where $\Lambda _t$, $\Lambda _1$, $\Lambda _2$ are defined according to (11), (15), (16), respectively. Within an optimized parallel-in-time solver, we can replace line 5 in Algorithm 1 with the procedure Algorithm 2 and calculate the inverse action at each time step in parallel.

## Numerical Results

In this section, we showcase the performance of the set of preconditioners (8) with different solution methods for the system (13) arising at each time step. We consider two (2+1)-dimensional test problems, one of which is the standard periodic problem ($\textrm{MFG}_{\mathbb {T}^{d}}$) with crowd aversion, and the other is the Neumann boundary problem ($\text {MFG}_{\Omega }$) with a terminal penalty on the density. We generally consider grid resolutions in the range $N_x\in \{16,32,64,128\}$, and for $N_y=N_x$ and $N_t=8N_x$, the size of the system (6) is $8N_x^3$, hence increasing by a factor $2^3$ when the grid spacing is halved. The largest problem we consider has approximately 16.8 million unknowns arising at each CP iteration when just accounting for the system (6). We have in total five times this number of unknowns (*m*, *w*) to be determined at each CP iteration, i.e., up to 83.9m unknowns.

We describe here briefly the algorithm setup. In the accelerated CP [30], the parameters are updated at each CP iteration. In the first iteration, the primal and dual step sizes are both set to $\tau _0 = \sigma _0 = 1$. At CP iteration ℓ+1, we first calculate the extrapolation parameter

$$\begin{aligned} \theta _{\ell +1} = \frac{1}{\sqrt{1+2\gamma \tau _{\ell }}}, \end{aligned}$$

where the strong convexity constant γ of the objective is set at the beginning of the algorithm, and hence update the primal and dual step sizes

$$\tau _{\ell +1} = \theta _{\ell +1}\tau _{\ell }, \quad \sigma _{\ell +1} = \frac{\sigma _{\ell }}{\theta _{\ell +1}}.$$

Note that the CP parameters satisfy σ>0, τ>0, $\theta \in [0, 1]$, and $\sigma \tau \le 1$ at all CP iterations. Although the accelerated CP is only guaranteed to converge under strong convexity, we observed that a small constant $\gamma \sim 10^{-3}$ leads to good convergence in practice. The CP algorithm terminates when the following criterion is satisfied,

$$\begin{aligned} r = \Vert m_{\ell +1} - m_{\ell }\Vert \le \texttt {CPtol}, \end{aligned}$$

i.e., when *r*, the change in *m* at CP iteration ℓ+1 compared to the previous iteration, falls below a given tolerance CPtol. In general, we set $\texttt {CPtol}=10^{-4}\Vert m_0\Vert$, noting that $m_0$ is a probability distribution and hence cannot be identically zero. In test cases that use a preconditioner for the inner solve, we use CG with relative tolerance set according to the rule

$$\begin{aligned} \texttt {CGtol}=\min \{10^{-4}, \max \{10^{-6}, 10^{-4}r\}\}{,} \end{aligned}$$

multiplied by the norm of the initial residual.

The solution method is implemented in *Python 3.10* using *SciPy* [70] for standard linear algebra operations and solvers. The solution method, including the application of the preconditioner, is parallelized using the *mpi4py* [71] library to ensure proper communication within the parallel infrastructure. We use 32 CPU cores allocated by an HPC cluster scheduler where different runs might use different processor models: *Intel Xeon Gold 5218 (2.30 GHz)*, *Intel Xeon Gold 6248 (2.50 GHz)*, or *AMD EPYC 8534P (64-core)*.

### Problem 1: Crowd Aversion with Trigonometric Cost Terms

**Fig. 2:**
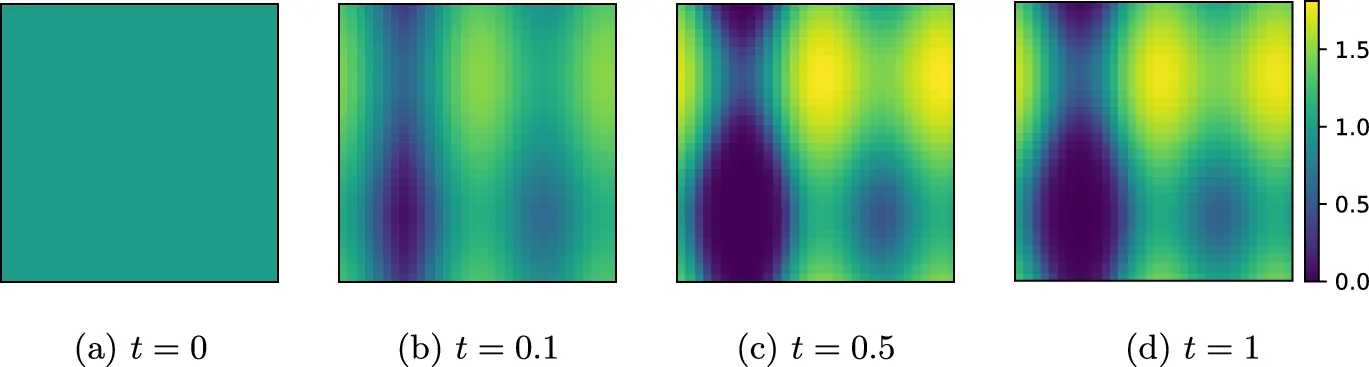
Solution to Problem 1 ($\textrm{MFG}_{\mathbb {T}^{d}}$), with $N_x=N_y=32$, $N_t=5N_x$, and ν=0.01

For this example, we simulate crowd aversion by setting

$$ f(x, y, m) = \frac{1}{2} \left( m^2-\sin (2\pi y)-\sin (2\pi x)-\cos (4\pi x)\right) $$

and take the Hamiltonian *H* given by (1) with $q^{\prime }=2$. We set the initial density $$m_0=1$$, take g≡0, impose periodic boundary conditions, and set $$\gamma =5\cdot 10^{-4}$$. The solution *m* is shown at four different time steps in Fig. 2, illustrating the dynamic behaviour of the solution. We observe similar behaviour to [21] where the dynamic solution is close to the solution of the steady problem apart from the initial and final phase. Towards the end of the time interval, the cost function pushes the solution away from the stationary one, an observation known as the *turnpike phenomenon* [21].

Table 1 shows the performance of the preconditioner (8) for four problem sizes $N_tN_x^2$, with $N_t=8N_x$, and viscosity ν=0.01. Columnwise, the results are given in terms of CP iterations (“CP iter”), average number of inner iterations (“Avg CG”), and CPU time in seconds including solver setup time (“Time [s]”) for two choices of preconditioner in time (8) (DST-I or DCT-VIII). The rows titled “LU” provide a benchmark in the form of an exact solve using LU decomposition with the *SciPy SuperLU* solver pre-computed at the start of the CP algorithm. Note that the DST/DCT columns are irrelevant in this case. Evidently, the LU decomposition cannot be computed for the two larger problems due to lack of memory with 32 GB (“NS”). Next, we employ the parallel-in-time preconditioner (8) and an LU solve for the system arising at each time step (13) (“PinT + LU”). We also employ a *PyAMG Smoothed Aggregation* solver [72–74] for the term (13), with one (“PinT + 1 *V*-cycle”) and two (“PinT + 2 *V*-cycles”) *V*-cycles, 3–4 grid levels (with a higher number providing no improvement), and no requirement for the relative residual norm tolerance. Lastly, we employ a fully recursive preconditioner using a DFT decomposition in space (18). For all three approaches, the solvers for the systems arising at each time step (13) are initialized at the start of the CP algorithm and called at each CG iteration.

The CP iteration numbers are identical for all solution methods, and the CG iteration numbers similarly remain stable for all problem sizes and solution methods, indicating robustness. The parallel-in-time preconditioner with a DCT decomposition in time offers the best results for most problems. Note that the inner LU decomposition and the recursive decomposition are exact (at least in exact arithmetic), hence we obtain the same inner iteration numbers. Using a multigrid method for the system arising at each time step, the inner iteration numbers increase slightly, however remaining contained with at most 53 inner iterations on average for the DST decomposition in time, and 25 for the DCT decomposition. Using the recursive linear algebra method gives an ideal approximation for rectangular grids, and it leads to a decrease in both inner iteration numbers and CPU time. The problem is solved for 16.8m variables, remaining robust with respect to inner iteration numbers and CPU time, regardless of the choice of solver for the system arising at each time step.

**Table 1:** Numerical results for the parallel-in-time preconditioner with various types of inner solves vs. an exact outer solve, for a periodic problem with viscosity ν=0.01

	$N_x$	Prob. size	CP iter	Avg CG	Time [s]	Avg CG	Time [s]
				(DST)		(DCT)	
LU	16	32 768	77	–	9.79	–	–
	32	262 144	67	–	611.38	–	–
	64	2 097 152	NS	–	NS	–	–
	128	16 777 216	NS	–	NS	–	–
PinT	16	32 768	77	11.8	10.78	2.4	29.14
+ LU	32	262 144	67	17.4	135.18	2.4	63.35
	64	2 097 152	70	26.1	325.17	2.4	266.22
	128	16 777 216	68	38.5	2 906.72	2.4	1 695.67
PinT	16	32 768	77	19.2	12.90	6.5	8.20
+ 1 *V*-cycle	32	262 144	67	26.0	67.08	8.8	32.40
	64	2 097 152	70	35.7	326.67	12.9	283.13
	128	16 777 216	68	52.5	2 519.46	24.2	2 688.48
PinT	16	32 768	77	17.6	13.69	4.3	8.13
+ 2 *V*-cycles	32	262 144	67	24.6	54.71	6.1	35.24
	64	2 097 152	70	34.1	367.92	8.9	268.77
	128	16 777 216	68	47.7	2 822.89	16.5	2 621.17
PinT	16	32 768	77	11.8	10.21	2.4	6.27
+ recursive	32	262 144	67	17.4	38.22	2.4	31.51
	64	2 097 152	70	26.1	268.66	2.4	234.78
	128	16 777 216	68	38.5	2 192.06	2.4	1 680.79

The performance of the fully recursive preconditioner is shown for different viscosities in Table 2 in terms of average performance (“Avg DST/DCT perf”), i.e., the average CG iteration numbers across all CP iterations (“CG iter”) and the average CPU time for one CG iteration (“Time [s]”). The best performance in terms of inner iterations and CPU time is indicated in bold for each problem size. Both preconditioners show robustness with respect to the viscosity and problem sizes, and the CPU times are again similar for both. While there is an increase in outer iterations for smaller viscosity as the problem becomes harder to solve, the CG iteration numbers decrease. This is particularly the case for the DCT decomposition, as noted in bold, as it becomes exact for zero viscosity. Nevertheless, the DST preconditioner is faster in terms of CPU time per CG iteration, also noted in bold. This is partly due to the custom DCT-VIII implementation, which introduces an additional computational overhead. Performance could likely be improved with a more optimized version, such as a *C++* implementation. As shown in Table 1, this highlights a trade-off: although DCT may reduce iteration counts, the readily available DST preconditioner offers comparable total runtime due to its more efficient execution and smaller memory requirement.

**Table 2:** Numerical results for the recursive parallel-in-time preconditioner, for a periodic problem with different viscosities

				Avg DST perf		Avg DCT perf	
ν	$N_x$	Prob. size	CP iter	CG iter	Time [s]	CG iter	Time [s]
1	16	32 768	20	7.4	**0**.**01**	**4**.**7**	0.03
	32	262 144	20	8.9	**0**.**06**	**4**.**9**	0.11
	64	2 097 152	20	11.6	**0**.**31**	**4**.**8**	1.37
0.1	16	32 768	21	13.9	**0**.**02**	**3**.**9**	0.04
	32	262 144	21	19.5	**0**.**09**	**3**.**9**	0.13
	64	2 097 152	21	27.4	**0**.**14**	**4**.**0**	1.60
0.01	16	32 768	77	11.8	**0**.**01**	**2**.**4**	0.03
	32	262 144	67	17.8	**0**.**03**	**2**.**4**	0.20
	64	2 097 152	70	26.1	**0**.**15**	**2**.**4**	1.40
0.001	16	32 768	69	13.1	**0**.**03**	**2**.**0**	0.15
	32	262 144	56	17.8	**0**.**03**	**2**.**0**	0.24
	64	2 097 152	72	23.3	**0**.**16**	**2**.**0**	1.69

### Problem 2: Gaussian Final Target State

The second example we implement is the Neumann problem ($\text {MFG}_{\Omega }$) with $\Omega =[-1/2,1/2]\times [-1/2,1/2]$. We set a final penalty $g(x,y,m)=\frac{1}{\beta }(m-\bar{m})$, where $\bar{m}$ is the target density, and β is a parameter determining the importance of reaching the target state, with smaller values of β promoting convergence to the target. We set $\beta =10^{-3}$, noting that the term *g* on the discrete level is weighted by $1/(\beta \Delta t)$, and the initial and target densities are Gaussian functions set to, respectively,

$$\begin{aligned} m_0=3\exp ^{-2^7\left( \left( x+\frac{1}{4}\right) ^2+\left( y-\frac{1}{4}\right) ^2\right) }, \quad \bar{m}=3\exp ^{-2^7\left( \left( x-\frac{1}{4}\right) ^2+\left( y+\frac{1}{4}\right) ^2\right) }. \end{aligned}$$

**Fig. 3:**
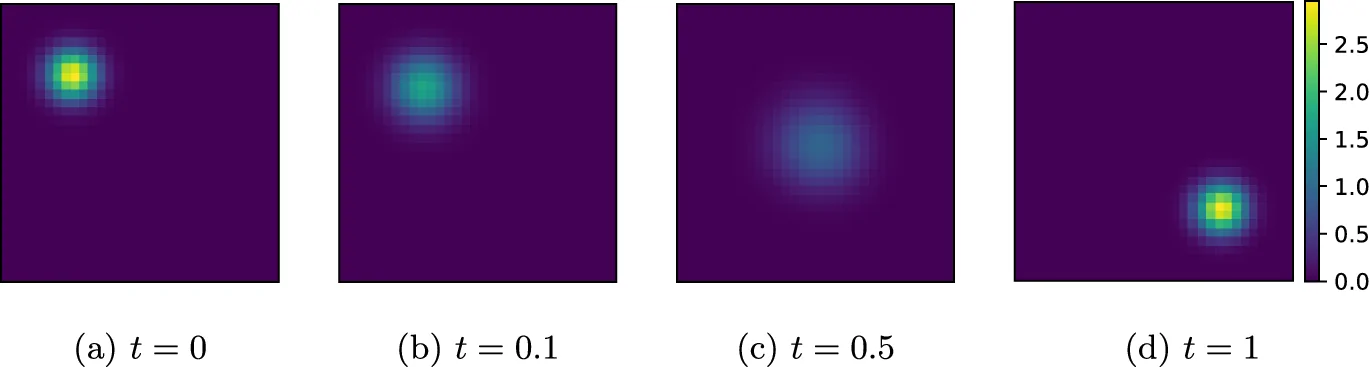
Solution to Problem 2 ($\text {MFG}_{\Omega }$) with a Gaussian target density, and $N_x=N_y=32$, $N_t=5N_x$, ν=0.01, and $\beta \Delta t=10^{-5}$

We also consider the quadratic Hamiltonian (1) with $q^{\prime }=2$, and set f(x,y,m)≡0. The CP parameter γ is set to $$\gamma =5\cdot 10^{-3}$$. The solution is shown in Fig. 3 for viscosity ν=0.01.

We repeat the same test as in Table 2, now for the Neumann problem, using an exact DCT-II decomposition in space (18), as shown in Table 3. This problem is significantly harder to solve, hence more outer iterations are needed to solve the problem and longer CPU times. Note, however, that the number of inner iterations is similar to those shown for the periodic problem in Table 2, indicating robustness of the recursive preconditioners. The largest system we apply the preconditioner to has 2.1m variables, with 5 times this many unknowns at each CP iteration.

**Table 3:** Numerical results for the fully recursive parallel-in-time preconditioner, applied to the Neumann problem with different viscosities

ν	$N_x$	Prob. size	CP iter	Avg CG	Time [s]	Avg CG	Time [s]
				(DST)		(DCT)	
1	16	32 768	109	11.5	9.59	5.2	6.89
	32	262 144	118	15.3	46.49	5.4	28.49
	64	2 097 152	124	20.6	252.98	5.0	198.45
0.1	16	32 768	476	22.0	54.98	4.2	23.90
	32	262 144	549	30.3	233.01	4.0	139.83
	64	2 097 152	674	40.5	1 789.48	4.0	1 219.40
0.01	16	32 768	354	21.6	38.84	3.8	16.33
	32	262 144	410	29.2	154.15	3.8	83.00
	64	2 097 152	532	40.3	1 058.02	3.6	641.61
0.001	16	32 768	351	20.3	33.68	2.6	16.08
	32	262 144	404	25.0	132.27	2.4	61.53
	64	2 097 152	592	30.8	949.92	2.3	670.74

**Table 4:** Numerical results for the parallel-in-time preconditioner and an AMG inner solver, applied to the Neumann problem with different viscosities

ν	$N_x$	Prob. size	CP iter	Avg CG	Time [s]	Avg CG	Time [s]
				(DST)		(DCT)	
1	16	32 768	109	50.0	38.38	48.6	42.87
	32	262 144	118	95.7	184.93	99.9	197.13
	64	2 097 152	122	215.3	1 569.09	220.5	1 877.07
0.1	16	32 768	476	27.7	114.81	16.3	73.47
	32	262 144	549	41.2	495.64	33.3	396.83
	64	2 097 152	674	75.9	3 796.97	63.9	3 984.10
0.01	16	32 768	354	28.8	76.75	6.0	25.41
	32	262 144	410	38.1	291.08	7.5	106.54
	64	2 097 152	532	46.6	1 942.38	9.4	961.39
0.001	16	32 768	351	28.4	76.67	4.7	20.90
	32	262 144	404	36.0	276.09	5.1	82.59
	64	2 097 152	592	44.3	1 929.73	5.3	751.08

Next, we repeat the same test using an AMG solver for the system arising at each time step, shown in Table 4. The AMG method performs very well for small viscosity but relatively poorly for large viscosity, whereas the recursive preconditioner shows stable performance for any viscosity in Table 3. However, since the overall solution time of AMG is comparable to that of the fully recursive approach, at least for small viscosity, its practical advantages should be carefully considered. In particular, AMG benefits from mature open-source implementations and naturally accommodates non-uniform grids and complex geometries. By contrast, the spatial recursive linear algebra approach is restricted to rectangular domains with finite-difference discretizations, but is found to be faster in that setting.

### Parallelization

To test the performance of the solution method with respect to scalability and parallelization, we perform a strong and weak scaling test. First, we run three fixed size problems to see if increasing the number of cores decreases the runtime. Second, we fix the proportional problem size in each core and increase the number of cores to see if the runtime remains constant. All parameters are set to the same values as those mentioned previously.

The parallelization can be described as follows: Generally all vectors are divided into temporal subvectors. The number of subvectors that need to be defined for each core is the total number of time steps divided by the number of cores, currently implemented without a possible remainder, i.e., $N_t$ must be divisible by the number of cores. Various subvectors need to be communicated across cores due to the time-stepping in (6) involving the previous and next time steps. Similarly, there are special conditions for the subvectors involving the first and final time step. Matrix and vector multiplications are only performed for those matrices and vectors specified for each core. The CG method is parallelized by computing matrix and vector multiplications and vector norms separately and gathering them whenever necessary. For the preconditioner, the DST or DCT transformation is applied in parallel by dividing the input vector of Algorithm 1 into *spatial* subvectors and hence performing the transformation. The vector is then redistributed in the time dimension, and the solution of *temporal* subproblems (13) is performed parallel-in-time. Lastly, the inverse transformation is applied spatially in parallel, and the output from CG is redistributed evenly to the cores in order to apply the remaining steps of CP (involving the proximal operators and the error norm) parallel-in-time. For the following scaling tests, we use a DCT decomposition in time and run exactly 100 iterations of the CP algorithm.

**Fig. 4:**
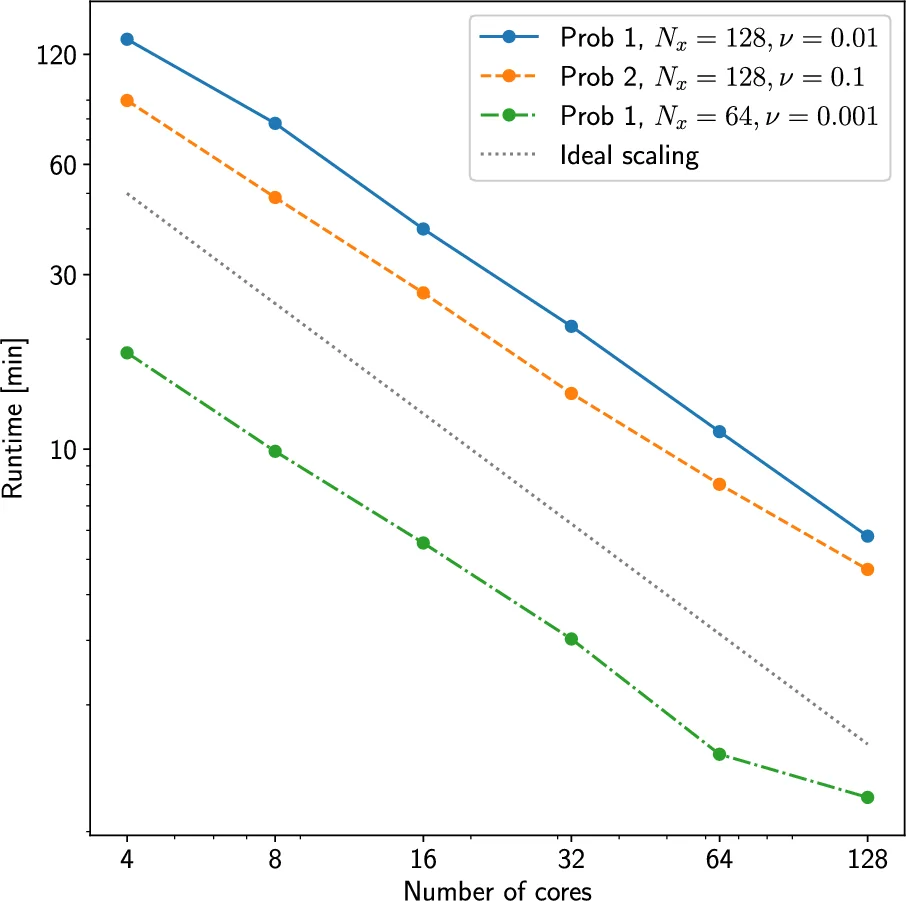
Strong scaling of the preconditioned method on a log–log scale, for three problem configurations

For the strong scaling test, we set $N_y=N_x$ and $N_t=4N_x$ and run three test problem configurations: (i) Problem 1, $N_x=128$, ν=0.01; (ii) Problem 2, $N_x=128$, ν=0.1; and (iii) Problem 1, $N_x=64$, ν=0.001. The two larger problems are allocated 8 GB of memory per core and the smaller one 6 GB per core, although actual usage showed that only 3 GB/core (1 GB/core for the smaller problem) was sufficient when using 4 cores, due to reduced communication buffer requirements. Figure 4 shows the strong scaling performance of the recursive preconditioner. Evidently, the runtime is approximately halved whenever double the amount of cores are available to the solver. The slight runtime overhead can be explained by increased communication and synchronization costs between processors as the problem size per processor decreases.

Table 5 shows the weak scaling of the recursive DCT preconditioner when the size of the subproblem sent to each core remains constant. The spatial refinement is set to either $N_x=N_y=32$ or $N_x=N_y=64$, and the viscosity is set to ν=0.01. The number of time steps $N_t$ is then set to 8 or 16 times the number of cores used for the smaller or larger problem, respectively. The two configurations are repeated for both spatial decompositions, i.e., the periodic (“Prob 1”) and the Neumann problem (“Prob 2”). Evidently, the runtime remains fairly constant across all problem instances. For the coarser grid, the runtime approximately doubles in the worst case when the problem size increases by a factor 32. For the finer grid, the runtime remains relatively unchanged when the problem size increases by a factor 32, indicating solid scalability, but the runtime is inevitably subject to randomness due to fluctuating load and resource contention on the shared supercomputing cluster. Note additionally that the runtime overhead of the Neumann problem shown in Table 3 is due to the number of CP iterations, and not the preconditioning, as Table 5 shows shorter runtime per CP iteration for the Neumann problem.

**Table 5:** Weak scaling of the preconditioned method with 100 CP iterations and ν=0.01, for varying $N_t$ and two spatial configurations

# Cores	$N_t$	Prob. size	Runtime [s]	
			Prob 1	Prob 2
$$N_x=N_y=32$$				
4	32	32 768	39.65	16.21
8	64	65 536	40.28	22.87
16	128	131 072	44.15	24.85
32	256	262 144	46.01	26.03
64	512	524 288	45.08	31.36
128	1024	1 048 576	50.59	34.42
$$N_x=N_y=64$$				
4	64	262 144	414.07	144.60
8	128	524 288	447.53	135.67
16	256	1 048 576	414.32	141.18
32	512	2 097 152	428.67	142.93
64	1024	4 194 304	408.46	163.20
128	2048	8 388 608	422.73	162.80

## Concluding Remarks

In this work, we introduced a novel preconditioning strategy for the efficient solution of linear systems arising in primal–dual methods applied to finite-difference discretizations of time-dependent variational MFG systems. Our approach leveraged parallel-in-time techniques based on DFTs, yielding a family of block-diagonalizable preconditioners that are well-suited for high-performance parallel implementations. This methodology yields a preconditioner that approaches the exact system in the zero-viscosity limit. We further presented efficient solvers for the systems arising at each time step, including recursive direct methods for structured grids with periodic and Neumann boundary conditions, and more general-purpose solvers for irregular grids. Numerical experiments confirm the efficiency and scalability of the proposed preconditioners on the individual linear systems, highlighting their potential for large-scale optimal control simulations.

Future work may explore extensions of this approach to other discretizations in time, such as the Jordan–Kinderlehrer–Otto scheme (JKO) time-stepping scheme [75], which has received recent attention in the literature as it has been applied to solve nonlinear Fokker–Planck equations by using, for instance, primal–dual algorithms [76]. Other possible extensions of our work include MFGs with non-local couplings [77], the MFG planning problem (where a target density is imposed within the constraints [25, 78]), and MFGs with congestion [79]. The solution method can be improved by including accelerators from the popular primal–dual approach for linear programs, extending the parallel implementation to usage on GPUs, or applying preconditioned iterative solvers within different outer iterative methods such as ADMM or Newton’s method.

## Data Availability

This paper has no associated data.

## Code Availability

The code used for this paper is available on GitHub.
